# Repurposing flubendazole for glioblastoma ferroptosis by affecting xCT and TFRC proteins

**DOI:** 10.1111/jcmm.70188

**Published:** 2024-11-14

**Authors:** Wei Teng, Yuanguo Ling, Niya Long, Wu Cen, Hongzhi Zhang, Lishi Jiang, Jian Liu, Xingwang Zhou, Liangzhao Chu

**Affiliations:** ^1^ Department of Neurosurgery The Affiliated Hospital of Guizhou Medical University Guiyang Guizhou China; ^2^ Department of Clinical Medicine Guizhou Medical University Guiyang Guizhou China; ^3^ Department of Neurosurgery, Guizhou Provincial People's Hospital Guiyang Guizhou China

**Keywords:** ferroptosis, Fubendazole, glioblastoma, glioblastoma stem cell

## Abstract

New uses of old drugs hold great promise for clinical translation. Flubendazole, an FDA‐approved antiparasitic drug, has been shown to target p53 and promote apoptosis in glioblastoma (GBM) cells. However, its damaging mechanism in GBM remains elusive. Herein, we explored the ferroptosis‐inducing ability of flubendazole on GBM cells. After treating glioma cell lines U251 and LN229 with the flubendazole (DMSO <1‰), cell viability was inhibited in a concentration‐dependent manner (IC_50_ for LN229 = 0.5331 μM, IC_50_ for U251 = 0.6809 μM), attributed to the induction of ferroptosis, as evidenced by increased MDA levels, accumulation of ROS and lipid peroxides, change in mitochondrial membrane potential and structure. Protein analysis related to ferroptosis showed upregulation of TFRC, DMT1 and p53, alongside downregulation of xCT, FHC and GPX4 (*p* < 0.05). All‐atom docking studies demonstrated that flubendazole bound closely with xCT, and TFRC, validating its role in inducing glioma ferroptosis via modulation of these proteins. Notably, flubendazole could damage the glioblastoma stem cells (GSC) that are typically resistant to other therapies, thereby possessing advantages in stopping glioma recurrence. This study delved into the mechanisms of flubendazole‐induced ferroptosis in glioma, broadening its application and providing new ideas for new uses of other old drugs.

## 1 INTRODUCTION

Glioblastoma, known as glioblastoma multiforme (Glioblastoma Multiforme, GBM), is one of the most complex and malignant tumours of the central nervous system, characterized by strong invasion, obvious ischemic necrosis and extensive microvascular hyperplasia.^1^, ^2^, ^3^ Despite the achievements in GBM treatments including surgery, radiotherapy and chemotherapy, the prognosis for GBM patients remains poor due to drug resistance and the presence of glioblastoma stem cells (GSC),^4^, ^5^, ^6^ which contribute to GBM progression and recurrence. Therefore, there is an urgent need to develop new therapeutic strategies or identify effective drugs to improve outcomes for GBM treatment.

Ferroptosis is a newly recognized type of programmed cell death that is iron‐dependent and produces harmful free radicals, leading to damage to cell membranes, oxidation and breakage of DNA and ultimately cell death.^7^ Importantly, previous studies have demonstrated that ferroptosis could act effectively on drug‐resistant cancer cells, making the induction of ferroptosis a promising anti‐GBM approach.^8^, ^9^, ^10^ In the last decades, much efforts have been devoted to developing novel ferroptosis‐inducing drugs but most of them are still far from clinical trials. In comparison, starting with an old drug to discover a new drug is often more fruitful, as their pharmacokinetics and safety profiles are already well‐established, making them more promising for clinical trials.

Flubendazole, an FDA‐approved anthelmintic, has been recently reported to inhibit glioma proliferation by inducing apoptosis via p53‐mediated G2/M arrests. However, given the diverse pathway associated with p53,^11^ we hypothesized that the anti‐GBM effect of flubendazole is not solely due to its pro‐apoptotic properties, and that the mechanism on glioma has not been fully elucidated. Interestingly, we found evidence in several publications suggesting that flubendazole may also influence ferroptosis.^12^, ^13^ To clarify this issue, in this study, we explored the ferroptosis‐inducing ability of flubendazole in GBM. We examined cell morphology, mitochondrial morphology, cell viability, cell cycle and apoptosis. In addition, we performed small‐molecule‐protein docking on proteins potentially targeted by flubendazole to understand the mechanisms underlying its anticancer effects, which is crucial for pharmacological analysis. Furthermore, we evaluated the effect of flubendazole on glioma stem cells, in an attempt to find methods to prevent glioma recurrence and metastasis.

## 2 MATERIALS AND METHODS

### 2.1 Cell culture and reagents

Human GBM cell lines LN229 and U251 were purchased from Procell (China). Cell cultures were grown at 37°C with 5% CO_2_ in a humidified atmosphere, in DMEM/high glucose medium (Gibco, USA) supplemented with 10% foetal bovine serum (Gibco, USA). Glioma stem cells (GSC‐1 and GSC‐2) were derived by U251 and LN229, and were cultured at 37°C with 5% CO_2_ in a humidified atmosphere, using DMEM/F12 glutamine medium (Gibco, USA) supplemented with 2% B27, 20 μM FGF and 20 μM EGF (Gibco, USA).^14^ Flubendazole, Ferrostatin‐1 (Fer‐1) and Liproxstatin‐1 (Lip‐1) were purchased from MCE (China). Rabbit polyclonal antibody against transferrin receptor (TFRC, AF5343, 1:1000), rabbit anti‐divalent metal transporter‐1 polyclonal antibody (DMT1, DF12740, 1:1000), rabbit polyclonal anti‐ferritin heavy chain antibody (FTH, DF6278, 1:1000), rabbit polyclonal antibody against system Xc^−^ (xCT, DF12509, 1:2000), rabbit anti‐p53 polyclonal antibody (p53, AF0879, 1:2000), rabbit anti‐glutathione peroxidase‐4 polyclonal antibody (GPX4, DF6701, 1:1000) were purchased from Affinity Biosciences (China).

### 2.2 Malondialdehyde (MDA) Content Assay

Malondialdehyde (MDA) Content Assay was purchased from Solarbio (Beijing, China). GSCs, U251 and LN229 cells were collected into centrifuge tubes. After centrifugation, the supernatant was discarded. Cell lysate (1 mL per 5 million cells) was added, and the mixture was placed on ice to react. Then, 500 μL of MDA extraction agent was added to each sample, followed by centrifugation at 8000 *g* at 4°C for 10 min. The supernatant was collected and kept on ice for testing. Next, a new EP tube was prepared, and the MDA test working solution, reagent III and the previously collected supernatant were added according to the kit instructions. The mixture was incubated in a 100°C water bath for 60 min, then cooled in an ice bath, and centrifuged at room temperature for 10 min at 10,000 *g*. A total of 200 μL of the supernatant was transferred to a trace glass cuvette or a 96‐well plate to measure the absorbance at 532 nm and 600 nm. The absorbance change was calculated as follows: ΔA532 = A532 (sample)−A532 (blank), ΔA600 = A600 (sample)−A600 (blank) and ΔA = ΔA532−ΔA600. The MDA concentration (nmol/10^4^ cells) was calculated using the formula: MDA (nmol/10^4^ cell) = [ΔA × V_total volume of the reaction system_ ÷ (ε × d) × 10^9^] ÷ (500 × Vsample ÷ Vextract) × F = 0.1075 × ΔA × F) where ε is the MDA mole absorbance coefficient (1.55 × 10^5^ L/mol/cm), d is the optical diameter of the 96‐well plate (0.6 cm), F is the dilution factor, multiplied by the dilution factor for hyperlipidemia or lipid samples (Here F=1).

### 2.3 Flow cytometry

Cell apoptosis was investigated using flow cytometry. According to the manufacturer's instructions, cell apoptosis was determined using the Annexin V‐FITC/PI apoptosis kit, and cell cycle analysis was performed using the Cell Cycle Staining Kit (Both kits were purchased from MULTI SCIENCES, China). For flow cytometry, the cell suspension was examined using a BD FACSLyric™ flow cytometer (BD Biosciences, USA). The flow cytometry data were analysed with BD FACSuite RUO software (BD Biosciences, USA). In the flow cytometry apoptosis plots, the first quadrant represented necrotic cells, the second quadrant represented late apoptotic cells, the third quadrant represented normal cells, and the fourth quadrant represented early apoptotic cells.

### 2.4 Lipid peroxidation analysis

All ROS analyses were performed with the fluorogenic probe DCFH‐DA using a Reactive Oxygen Species Assay Kit (Beyotime Biotechnology, S0033S, China). The DCFH‐DA (10 mM) was diluted 1000‐fold in serum‐free culture medium to a final concentration of 10 μM. After removing the cell culture medium, the diluted DCFH‐DA (10 μM) was added to the cells to fully cover them, ensuring no less than 1 mL per well in a six‐well plate. The cells were incubated in a 37°C cell incubator for 20 min, followed by washing three times with serum‐free culture medium to thoroughly remove residual DCFH‐DA. Then, the stained cell climbing slices were imaged using a fluorescence microscope (Leica, Germany).

Lipid ROS analysis was performed using the C11 BODIPY 581/591 lipid peroxidation fluorescent probe (MK Bio, China). Cells were incubated with C11 BODIPY 581/591 (2 μM in DMEM) for 20 min, followed by imaging with a fluorescence microscope. Excitation was performed at 488 nm and 565 nm, with detection of emitted light at 505–550 nm and >580 nm. C11 BODIPY 581/591 is a hydrophilic dye that accumulates in membranes. Upon oxidation, the dye's maximum emission wavelength shifts from 590 nm to 510 nm, while retaining lipophilicity, thereby reflecting the level of lipid peroxidation in the membrane.

### 2.5 Western blotting

Proteins were harvested from cells using RIPA lysis buffer (Solarbio, Cat#R0010, China) containing phenylmethanesulfonylfluoride (PMSF, Solarbio, Cat#R0010, China). Equal amounts of protein were separated by sodium dodecyl sulphate‐polyacrylamide gel electrophoresis and transferred onto polyvinylidene difluoride (PVDF) membranes (BIO‐RAD, USA). The membranes were blocked with milk and incubated with primary antibodies overnight at 4°C. Subsequently, the membranes were incubated with secondary antibodies for 1.5 h at room temperature. Finally, the bands were visualized using a HyperSignal ECL kit (NCM, China). Immunoblot signals were measured using the ChemiDoc™ MP Imaging System (BIO‐RAD, USA), and the average band intensity was calculated.

### 2.6 Cell immunofluorescence

Cell climbing slices were fixed with 70% ethanol, permeabilized with 0.5% Triton, blocked with 5% goat serum, and incubated with GPX4 primary antibody (1:100) at 4°C overnight. The slices were then incubated with goat anti‐rabbit IgG H&L (Alexa Fluor® 488) (ab150077, Abcam, USA) for 1.5 h at room temperature. Stained cell slices were imaged using a fluorescence microscope (Leica, Germany).

### 2.7 Transmission electron microscope

LN229 and U251 cells were collected and fixed with 3% glutaraldehyde and 0.1 M phosphate‐buffered saline (PBS, pH 7.4) at 24 h. The cells were then post‐fixed in 2% osmium tetroxide in PBS solution for 1 h at room temperature. The morphology of the mitochondria was observed and photographed.

### 2.8 Fluorescence staining of cellular iron

FerroOrange (F374, Dojindo Molecular Technologies, China) was used to detect the cellular iron. Briefly, U251 and LN229 cells were stained with FerroOrange (1 μM) for 20 min, washed with PBS, and then imaged by fluorescence microscope. MitoTracker Red CMXRos (Invitrogen™ M7512, Thermo Fisher Scientific Inc., USA) was used to co‐stain with the FerroOrange.

### 2.9 Cell‐counting kit 8 (CCK‐8) assay

The growth and viability of the U251 and LN229 cells were determined using the cell counting kit 8(CCK‐8, APEx Bio, China) assay. Cells were seeded at a density of 2.0 × 10^4^ cells per well in growth medium in 96‐well tissue culture plates. After adding 10 μL of CCK‐8 solution to each well, the cells were incubated for 1 h. The absorbance of each well was measured using an enzyme‐linked immunosorbent assay reader (VARIOSKAN LUX, Thermo Scientific, USA).

### 2.10 Statistical analysis

All data were presented as the mean ± SD. Significant differences between the means of the two groups were assessed using Student's *t*‐test (All experiments were repeated three times). All statistical analyses were undertaken using Prism 9 software (GraphPad Software).

## 3 RESULTS

### 3.1 Flubendazole inhibits the viability of glioblastoma cells

**FIGURE 1:**
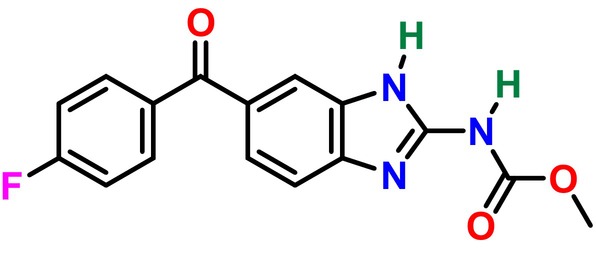
Flubendazole chemical structure. Picture source: PubChem (nih.gov).

**FIGURE 2:**
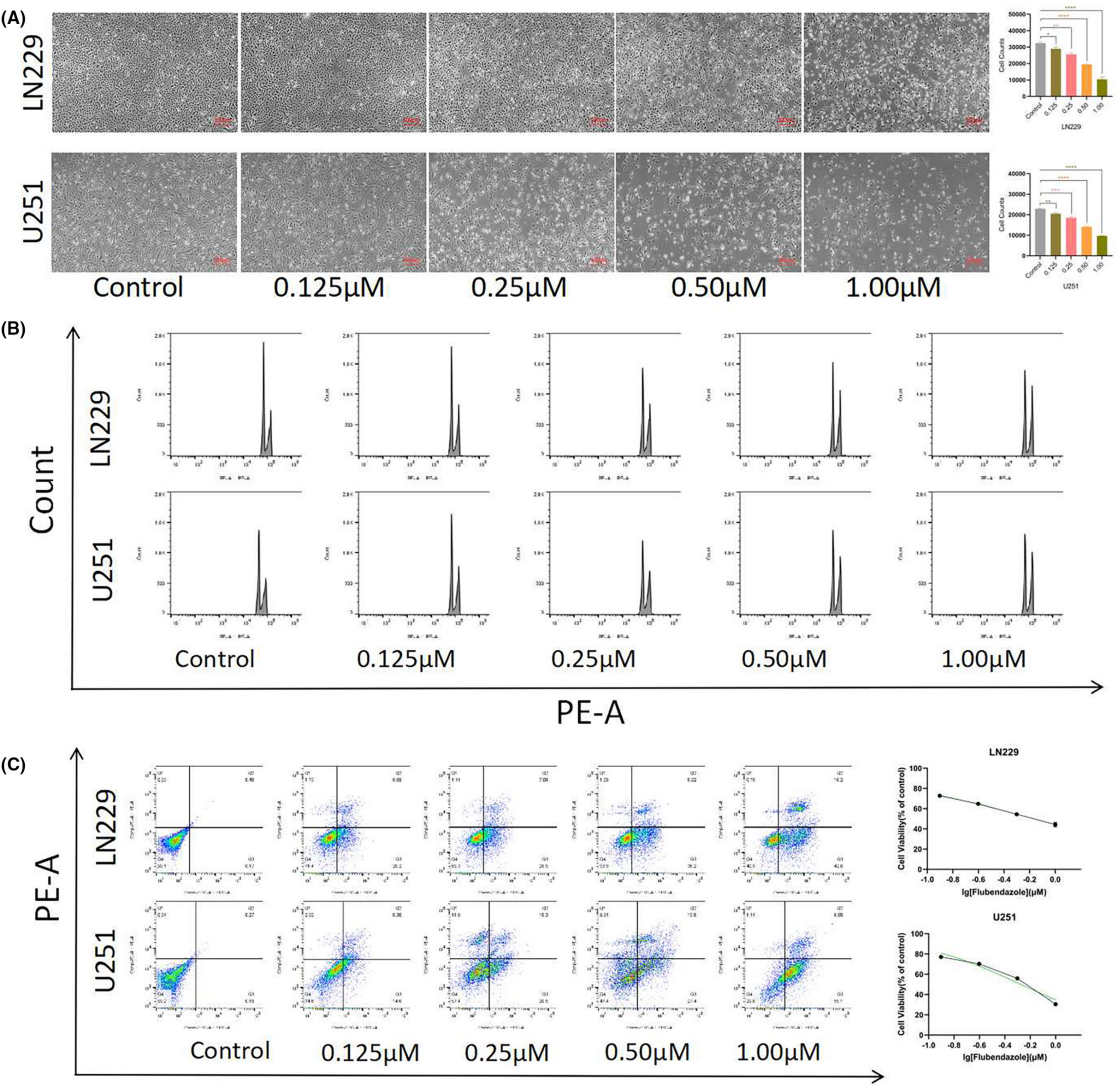
Flubendazole inhibited cell proliferation. (A) Flubendazole concentration gradient in LN229 and U251 cells for 24 h and decreased cell number(200×, 1:100 μm); (B) LN229 and U251 cells showed cell cycle arrest after Flubendazole treatment; (C) LN229 and U251 cells showed increased apoptosis after Flubendazole treatment; Calculated the apoptosis rate to obtain LN229 IC_50_ = 0.5331 μM, U251 IC_50_ = 0.6809 μM. (Flubendazole, 0, 0.125, 0.25, 0.5, 1.0 μM, 24 h; Student's *t*‐test was used. *p < 0.05, **p < 0.005, ***p < 0.001, ****p < 0.0001).

The anti‐proliferative activity of flubendazole (Figure 1) against glioma cells was first examined using glioma U251 and LN229 cell lines, similar to the study by Zhou X et al., which used SF‐268 and T98 cells.^15^ After treatment with Flubendazole, the growth of both LN229 and U251 glioma cell lines were inhibited with concentration‐dependence. Meanwhile, the cell morphology of both LN229 and U251 cells became disordered and blurred, further implying the anti‐glioma effect (Figure 2A). Flow cytometry demonstrated that the cell cycle was arrested in the G_2_/M phase (Figure 2B) and the proportion of apoptotic cells increased with increasing drug concentration, consistent with the above data (Figure 2C). The IC_50_ value, calculated based on the apoptotic data, were 0.6809 μM for U251 and 0.5331 μM for LN229 cells, respectively (Figure 2C). These data suggested that flubendazole effectively induced cytotoxicity in glioma cells.

### 3.2 Flubendazole induces peroxidation in glioma cells

**FIGURE 3:**
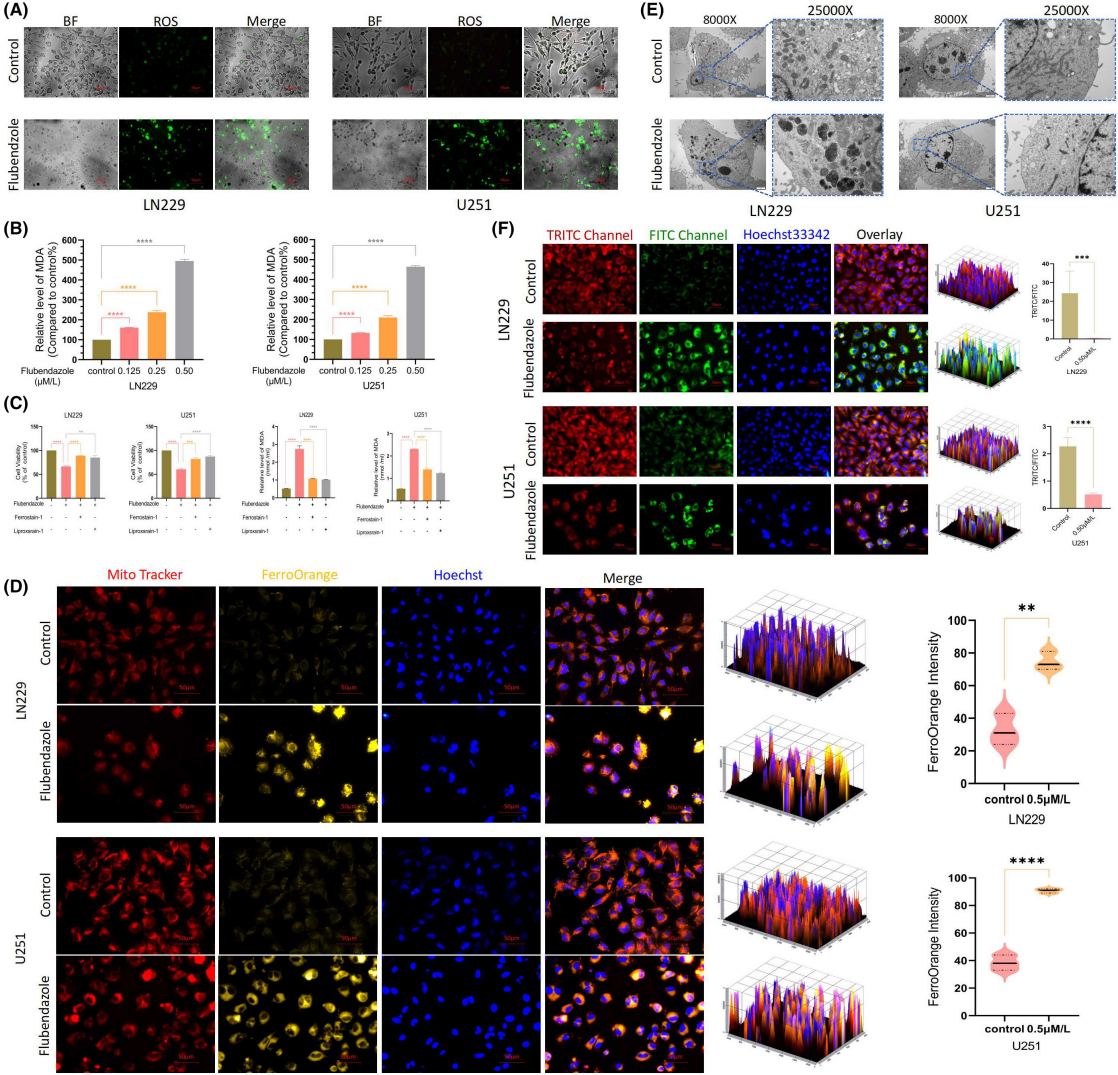
Flubendazole induced peroxide accumulation in glioblastoma cells. (A) After Flubendazole treatment with U251 and LN229 cells, ROS accumulation compared with control cells(400×, 1: 50 μm); (B) After the treatment with Flubendazole, a significant elevation of MDA was observed in both LN229 and U251 cells; (C) CCK‐8 survival was higher in U251 cells pre‐treated with iron‐death inhibitor for 12 h than in cells treated with direct drugs; U251 and LN229 cells pre‐treated with iron death inhibitors for 12 h had lower MDA content than cells treated with direct drugs. (D) After 24 h of drug treatment, LN229 and U251 cells showed ferrous accumulation, Enhanced orange fluorescence. (Flubendazole, 0.5 μM; 400×, 1: 50 μm). (E) U251 cells and LN229 cells treated with drugs for 24 h showed disappeared mitochondrial cristae, wrinkled mitochondria and deepening colour(8000×/25000×, 1: 500 nm/1: 2 μm); (F) Lipid peroxide detection of LN229 and U251 cells after drug treatment, which found that the drug‐treated LN229 oxidation state (green fluorescence) enhanced, reduced state (red fluorescence) decreased. (Flubendazole, 0.5 μM, 24 h; 400×, 1: 50 μm). (Flubendazole 0.5 μM, liproxstatin‐1 15 μM, Ferrostatin‐1 5 μM; repetitions per sample (*n* = 3), Student's *t*‐test was used. *p < 0.05, **p < 0.005, ***p < 0.001, ****p < 0.0001).

To delve into the cell damaging mechanisms, we then examined the condition of cellular oxidative stress. Preferentially, the cellular total ROS levels were measured via cell staining and the enhanced green fluorescence indicated that ROS levels were increased in both drug‐treated cell lines (Figure 3A). In addition, malondialdehyde (MDA) content, an indicator of lipid peroxidation,^16^ was measured following gradient administration of the drug. The results showed that MDA levels in both cell lines increased approximately fivefold compared to the control group (Figure 3B). The elevated MDA directly reflected the rate and intensity of lipid peroxidation that is fundamental for the occurrence of ferroptosis.^17^ Correspondingly, changes in lipid peroxidation were detected before and after drug treatment using a C11 BODIPY probe in both U251 and LN229 cells. An enhanced oxidation state was observed in the drug‐treated cells (Figure 3F), demonstrating the induction of ferroptosis. For further confirmation, the mitochondrial morphology was observed via electron microscopy. As shown in Figure 3E, the mitochondria in drug‐treated LN229 and U251 cells exhibited loss of cristae, wrinkling and deepened colour. Given that lipid peroxidation and the Fenton reaction involving free iron are fundamental to ferroptosis, we stained the cells with mitochondrial and iron ion probes to observe changes in intracellular iron levels. The results indicated an increase in intracellular iron ions after drug treatment, which co‐localized with the mitochondria (Figure 3D).

Additionally, we pre‐treated both cell lines with two ferroptosis inhibitors, liproxstatin‐1 and Ferrostatin‐1, for 12 h, followed by flubendazole treatment. After 24 h, cell viability was assessed using the CCK‐8 assay. The results showed that pre‐treatment with ferroptosis inhibitors effectively reduced cell death (Figure 3C). Meanwhile, MDA levels revealed that ferroptosis inhibitors partially rescued cell death caused by flubendazole, further demonstrating that their antitumor effect was associated with ferroptosis.

### 3.3 Flubendazole affects ferroptosis‐related proteins expression in GBM cells

**FIGURE 4:**
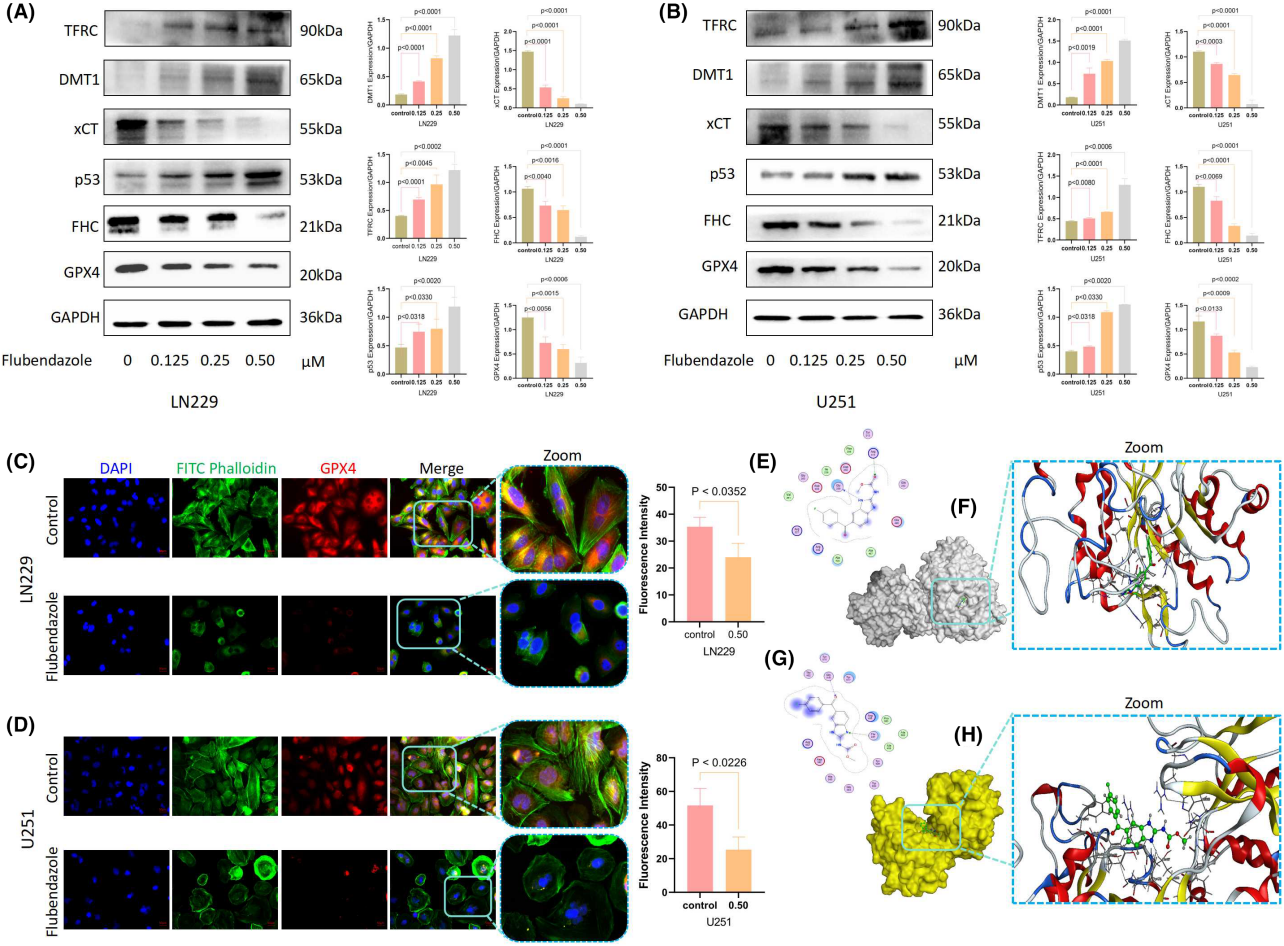
Flubendazole affects ferroptosis‐related protein expression in glioblastoma cells. (A, B) After the drug treatment, LN229 and U251 cells showed decreased expression of xCT, FHL and GPX 4, Accumulation of TFRC, DMT 1 and p53 (Flubendazole, 0, 0.125, 0.25, 0.5 μM, 24 h); (C, D) GPX4 depletion in U251 and LN229 cells treated with drugs (Flubendazole, 0.5 μM, 24 h; 400X, 1: 50 μm). (E–G) Flubendazole binds to two sites 229 Gly and 314 Arg in xCT protein. (2D diagram); (F, H) Flubendazole binds to two sites 457 Thr and 516 Gly in TFRC protein. (Student's *t*‐test was used.).

We next examined whether ferroptosis‐related proteins, particularly p53, TFRC, DMT1, xCT, FHC and GPX4, were affected by flubendazole. As presented in the protein imprinting and their semi‐quantitative analysis, the expression levels of p53, TFRC and DMT1 were upregulated, while those of xCT, FHC and GPX4 were downregulated after flubendazole treatment (Figure 4A,B). p53 is a key protein that regulates System Xc^−^; its activation would downregulate the expression of SLC7A11, inhibiting cysteine uptake. This reduction in cystine availability decreases the activity of cystine‐dependent glutathione peroxidase, promoting ferroptosis. Similarly, the overexpression of TFRC and DMT1 and the reduced expression of FHC would synergistically increase the susceptibility of iron pools and cells to ferroptosis. In addition, xCT is responsible for transporting cystine from the extracellular environment into the cell, which is then used to produce glutathione (GSH) to detoxify lipid ROS and prevent cell damage. Therefore, the depletion of xCT and GPX4 would lead to ROS accumulation and lipid peroxidation, resulting in cell membrane damage and triggering ferroptosis. Since GPX4 directly impacts ferroptosis, GPX4 expression was further explored by immunofluorescence. The reduced green fluorescence visually demonstrated that GPX4 expression in U251 and LN229 was inhibited after drug treatment (Figure 4C,D), corroborating the occurrence of ferroptosis. To identify the specific targets of flubendazole, we conducted all‐atom docking^18^ of multiple proteins involved in the ferroptosis pathway through MOE software. The results indicated strong binding affinities between flubendazole and proteins such as xCT and TFRC. Flubendazole was found to bind tightly to xCT at residues Gly229 and Arg314 (Figure 3E,F), and to TFRC at residues Thr457 and Gly516 (Figure 3G,H).

### 3.4 Flubendazole inhibits the CSC proliferation

**FIGURE 5:**
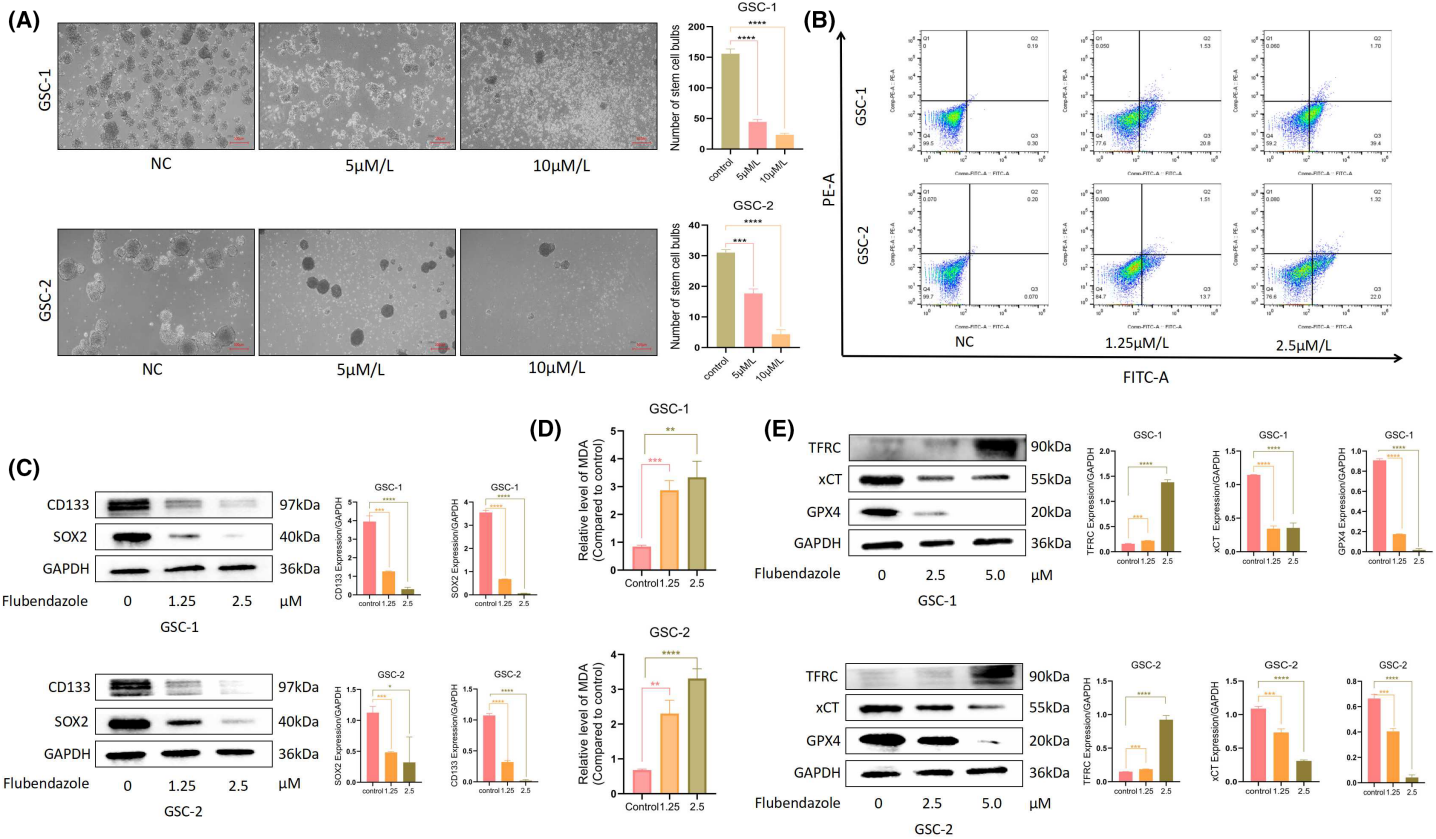
Flubendazole inhibited GSC proliferation and induced its apoptosis. (A) The gradient administration showed decreased GSC proliferation, decreased the number and size of neurospheres, and increased depolymerization of neurospheres and increased cell death (200X, 1:100 μm); (B) Apoptosis by flow cytometry revealed significant apoptosis in both GSCs after administration (Flubendazole, 24 h); (C) After treatment with Flubendazole, a decrease in CD133 and SOX2 was observed; (D) After Flubendazole treatment, there was a significant increase in MDA levels in the cells; (E) Following treatment with Flubendazole, TFRC showed a significant increase, while xCT and GPX4 decreased.(Student's *t*‐test was used. *p < 0.05, **p < 0.005, ***p < 0.001, ****p < 0.0001).

Previous studies have demonstrated that GSC, which are differentiated stem cells in GBM, exhibit high resistance to therapy and contribute significantly to tumour heterogeneity and growth.^19^, ^20^ Therefore, inhibiting GSC is particularly important for effective GBM treatment.^21^ We investigated whether flubendazole could inhibit GSC proliferation using a Stem‐cell globulation assay. In this study, GSC‐1 was derived from U251, while GSC‐2 was derived from by LN229. After gradient administration, flubendazole was found to inhibit GSC proliferation. At a concentration of 10 μM, stem cell bulbs were nearly undetectable, whith noting that 5 μM and 10 μM concentrations were excessively high (Figure 5A). We adjusted the flubendazole concentrations to 1.25 μM and 2.5 μM for subsequent experiments. Flow cytometry analysis revealed that GSC showed a tendency toward early apoptosis after drug treatment (Figure 5B). Furthermore, we assessed the expression levels of stemness markers CD133 and SOX2 inn GSC, and found a significant reduction in both of them, indicating that flubendazole effectively diminished the stemness of GSCs (Figure 5C). Additionally, MDA levels increased to a certain degree after drug treatment (Figure 5D), and changes were observed in ferroptosis‐related proteins. Specifically, TFRC showed a noticeable increase at a drug concentration of 2.5 μM, while xCT and GPX4 decreased (Figure 5E). This also suggests that flubendazole may reduce the recurrence of GBM by impairing GSC stemness and inducing ferroptosis.

## 4 DISCUSSION

**FIGURE 6:**
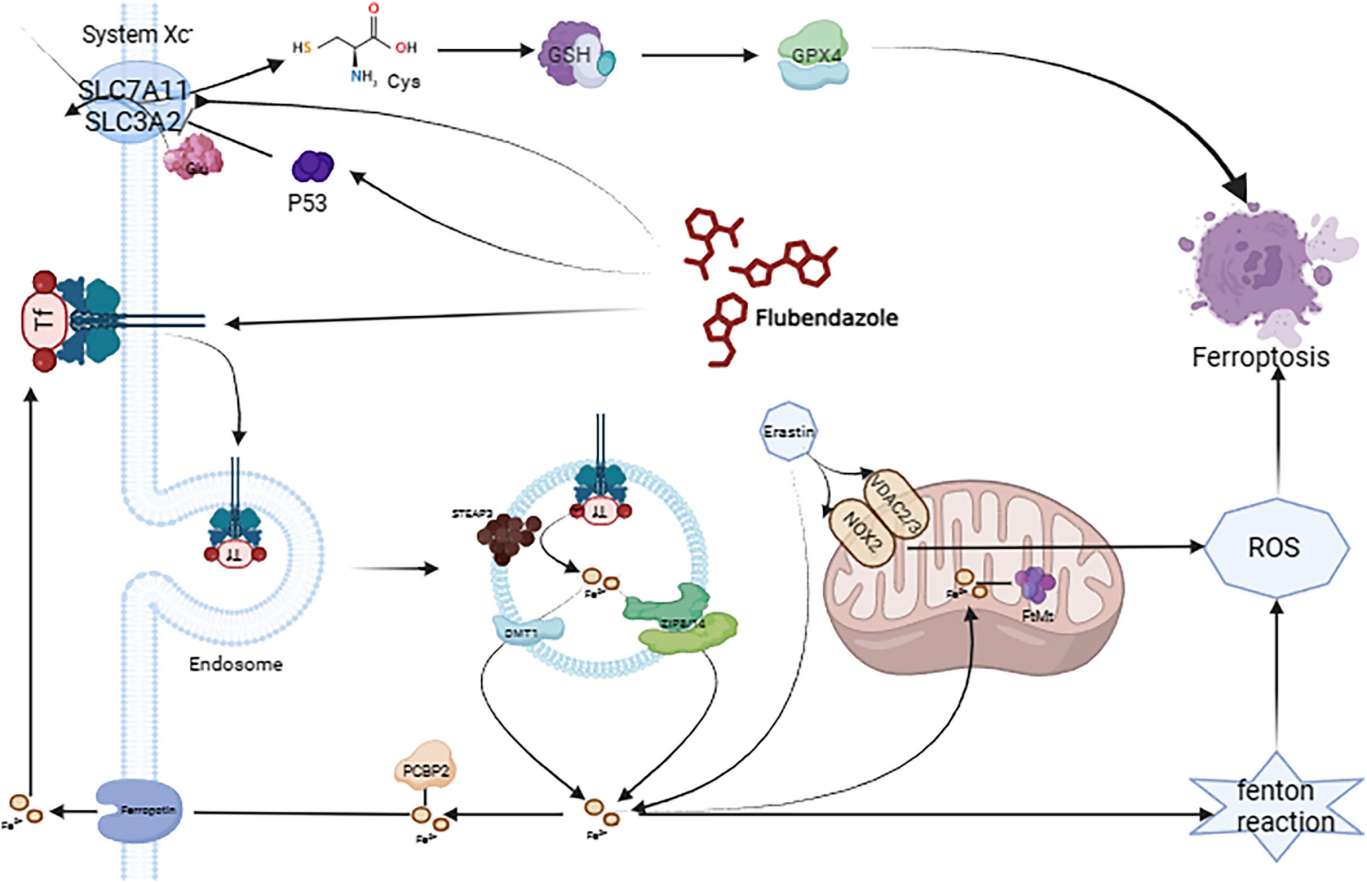
Schema of the mechanism of drug‐induced ferroptosis in glioma cells.

Unlike the traditional approach of developing new drugs from scratch, repurposing existing drugs can leverage existing pharmacokinetic and toxicity data, significantly reducing the time and cost associated with drug development.^22^ In this study, we explored the anticancer effects of flubendazole, a traditional antimalaria, in GBM and investigated its potential mechanisms of action. Our in vitro experiments revealed that flubendazole significantly inhibited the growth and migration of U251 and LN229 glioma cell lines. In addition to inducing apoptosis, we confirmed that flubendazole treatment also triggers ferroptosis. Mechanistically, flubendazole exerts its ferroptosis‐inducing ability by upregulating the expression of p53, TFRC and DMT1 and downregulating the expression of xCT, FHC and GPX4 (Figure 6).

Previous studies have shown that flubendazole can active p53 expression in GBM, thereby result in p53‐mediated G2/M arrest and pro‐apoptosis.^11^, ^12^ Given that p53 is involved in multiple pathways,^23^ we speculated that apoptosis might not be the only action of flubendazole in GBM. Potential anti‐GBM mechanisms of flubendazole could be more complicated. Recent reports demonstrated that flubendazole can regulate ferroptosis in castration‐resistant prostate cancer (CRPC) cells via p53/xCT/GPX4 axis.^12^ Our findings support this in GBM, showing that flubendazole triggers ferroptosis in GBM through p53 activation, characterized by mitochondrial disorder, increased ROS levels and enhanced lipid peroxidation.

Further, considering that ferroptosis is an iron‐ and ROS‐dependent form of cell death,^24^ we wondered whether flubendazole exerts its effects in GBM just via interacting with p53/GPX4 axis. It is well established that iron metabolism and lipid peroxidation signalling are both central mediators of ferroptosis.^26^ Excess iron would facilitate ferroptosis via Fenton reaction, with Fe^3+^ imported into cells via TFRC and reduced to Fe^2+^ in the endosome by STEAP3. DMT1 then mediates the release of Fe^2+^ into a labile iron pool and excess iron is stored in ferritin, including FHC.^25^ Thus, ferroptosis‐sensitive cells typically exhibit increased TFRC and decreased FHC levels. Interestingly, our data validated that the expression of TFRC and DMT1 was upregulated and FHC downregulated in glioma cells after flubendazole treatment. Moreover, we found that flubendazole binds tightly with xCT and TFRC, further revealing that the two central mediators of ferroptosis including p53/xCT/GPX4 and TFRC/DMT1/FHC signalling pathways are both accounting for ferroptosis‐inducing ability of flubendazole in GBM.

Additionally, GSC are known to drive tumour angiogenesis, enhance tumour invasion and spread, show high resistance to radiotherapy, and rapidly rebuild the tumour after conventional treatments, leading to the quick recurrence of glioma.^20^ Effective GSC injury therapy is crucial to improving glioma therapeutic outcomes. Our results demonstrated that flubendazole effectively inhibited GSC proliferation, with nearly no stem cell vesicles observed at a concentration of 10 μM, which may prevent the recurrence and improve therapeutic outcomes.

Despite these advancements, there may be some possible limitations in this study. For example, it may be necessary to extend the studies to other glioma cell lines or to primary cultures of patient glioma tissue to make the conclusions more general, owing to the limited number of cell lines used. Besides, while this study focused on the ferroptosis‐inducing mechanisms of flubendazole in GBM, the role of flubendazole in autophagy activation^26^ in GBM remains unexplored. What is more, ferroptosis is effective in those resistant cancers,^27^ synergies between flubendazole and other drugs could also be investigated in the future.

In conclusion, results from our study revealed that flubendazole can be repurposed as an anti‐GBM drug with both apoptosis and ferroptosis‐inducing capabilities. The mechanisms underlying its ferroptosis‐inducing effects involve both the p53/xCT/GPX4 and TFRC/DMT1/FHC pathways. Notably, GSC could also be inhibited by flubendazole for recurrence prevention. These data strongly indicate that repositioning flubendazole in glioma cells offers a promising therapeutic approach. The ability of flubendazole to target multiple pathways makes it a compelling candidate for further research and potential clinical use in glioma treatment.

## AUTHOR CONTRIBUTIONS

**Wei Teng:** Conceptualization (equal); data curation (equal); formal analysis (equal); investigation (equal); methodology (equal); project administration (equal); visualization (equal); writing – original draft (equal). **Yuanguo Ling:** Formal analysis (equal); funding acquisition (equal); investigation (equal); methodology (equal); writing – review and editing (equal). **Niya Long:** Funding acquisition (equal); writing – review and editing (equal). **Wu Cen:** Validation (equal). **Hongzhi Zhang:** Resources (equal). **Lishi Jiang:** Validation (equal). **Jian Liu:** Funding acquisition (equal). **Xingwang Zhou:** Formal analysis (equal); funding acquisition (equal). **Liangzhao Chu:** Conceptualization (equal); writing – review and editing (equal).

## FUNDING INFORMATION

This study was funded by National Natural Science Foundation of China (No. 82360493) to Xingwang Zhou; Guizhou Province Science and Technology Plan Project (Project) Qiankehe Foundation‐ZK[2023]General 360 to Niya Long; Qiankehe Foundation‐ZK[2023]General 362 to Liangzhao Chu; National Natural Science Foundation of China (NSFC), Affiliated Hospital of Guizhou Medical University(gyfynsfc‐2022‐25) to Niya Long; Science and Technology Fund project of Guizhou Provincial Health Commission(gzwkj‐2022‐09) to Liangzhao Chu; Science and Technology Fund project of Guizhou Provincial Health Commission. (gzwkj‐2023‐035) to Niya Long; Guizhou Science and Technology Plan Project (No. Qiankehe 2016 Support [2905]) to Jian Liu.

## CONFLICT OF INTEREST STATEMENT

All authors have no conflict of interest.

## Supporting information


Appendix S1:

Appendix S2:

Appendix S3:

Appendix S4:

Appendix S5:

Appendix S6:

Appendix S7:

Appendix S8:

Appendix S9:

Appendix S10.

Appendix S11.

Appendix S12.

Appendix S13.

Appendix S14.

Appendix S15.

Appendix S16.

Appendix S17.

Appendix S18.

Appendix S19.

Appendix S20.

Appendix S21.

Appendix S22.

Appendix S23.

Appendix S24.

Appendix S25.

Appendix S26.

Appendix S27.

Appendix S28.

## Data Availability

The original contributions presented in the study are included in the article. Further inquiries can be directed to the corresponding authors. All data are available at the first author or at the corresponding author.

## References

[1] Omuro A , DeAngelis LM . Glioblastoma and other malignant gliomas: a clinical review. JAMA. 2013;310(17):1842‐1850. doi:10.1001/jama.2013.280319 PMID: 24193082. 24193082

[2] Alexander BM , Cloughesy TF . Adult Glioblastoma. J Clin Oncol. 2017;35(21):2402‐2409. doi:10.1200/JCO.2017.73.0119 28640706

[3] Ma R , Taphoorn MJB , Plaha P . Advances in the management of glioblastoma. J Neurol Neurosurg Psychiatry. 2021;92(10):1103‐1111. doi:10.1136/jnnp-2020-325334 34162730

[4] Gimple RC , Bhargava S , Dixit D , Rich JN . Glioblastoma stem cells: lessons from the tumor hierarchy in a lethal cancer. Genes Dev. 2019;33(11–12):591‐609. doi:10.1101/gad.324301.119 31160393 PMC6546059

[5] Tabatabai G , Weller M . Glioblastoma stem cells. Cell Tissue Res. 2011;343(3):459‐465. doi:10.1007/s00441-010-1123-0 21253762

[6] Lah TT , Novak M , Breznik B . Brain malignancies: glioblastoma and brain metastases. Semin Cancer Biol. 2020;60:262‐273. doi:10.1016/j.semcancer.2019.10.010 31654711

[7] Jiang X , Stockwell BR , Conrad M . Ferroptosis: mechanisms, biology and role in disease. Nat Rev Mol Cell Biol. 2021;22(4):266‐282. doi:10.1038/s41580-020-00324-8 33495651 PMC8142022

[8] Chen X , Kang R , Kroemer G , Tang D . Broadening horizons: the role of ferroptosis in cancer. Nat Rev Clin Oncol. 2021;18(5):280‐296. doi:10.1038/s41571-020-00462-0 33514910

[9] Lei G , Zhuang L , Gan B . Targeting ferroptosis as a vulnerability in cancer. Nat Rev Cancer. 2022;22(7):381‐396. doi:10.1038/s41568-022-00459-0 Epub 2022 Mar 25. PMID: 35338310; PMCID: PMC10243716. 35338310 PMC10243716

[10] Hassannia B , Vandenabeele P , Vanden BT . Targeting ferroptosis to iron out cancer. Cancer Cell. 2019;35(6):830‐849. doi:10.1016/j.ccell.2019.04.002 31105042

[11] Jiang L , Kon N , Li T , et al. Ferroptosis as a p53‐mediated activity during tumor suppression. Nature. 2015;520(7545):57‐62. doi:10.1038/nature14344 25799988 PMC4455927

[12] Zhou X , Zou L , Chen W , et al. Flubendazole, FDA‐approved anthelmintic, elicits valid antitumor effects by targeting P53 and promoting ferroptosis in castration‐resistant prostate cancer. Pharmacol Res. 2021;164:105305. doi:10.1016/j.phrs.2020.105305 33197601

[13] Khachigian LM . Emerging insights on functions of the anthelmintic flubendazole as a repurposed anticancer agent. Cancer Lett. 2021;1(522):57‐62. doi:10.1016/j.canlet.2021.09.013 34520820

[14] Chen C , Jing W , Chen Y , et al. Intracavity generation of glioma stem cell‐specific CAR macrophages primes locoregional immunity for postoperative glioblastoma therapy. Sci Transl Med. 2022;14(656):eabn1128. doi:10.1126/scitranslmed.abn1128 Epub 2022 Aug 3. PMID: 35921473. 35921473

[15] Zhou X , Liu J , Zhang J , Wei Y , Li H . Flubendazole inhibits glioma proliferation by G2/M cell cycle arrest and pro‐apoptosis. Cell Death Dis. 2018;14(4):18. doi:10.1038/s41420-017-0017-2 PMC5841417 29531815

[16] Yi R , Wang H , Deng C , et al. Dihydroartemisinin initiates ferroptosis in glioblastoma through GPX4 inhibition. Biosci Rep. 2020;40(6):BSR20193314. doi:10.1042/BSR20193314 PMID: 32452511; PMCID: PMC7313443. 32452511 PMC7313443

[17] Song Q , Peng S , Che F , Zhu X . Artesunate induces ferroptosis via modulation of p38 and ERK signaling pathway in glioblastoma cells. J Pharmacol Sci. 2022;148(3):300‐306. doi:10.1016/j.jphs.2022.01.007 35177209

[18] Liu Y , Yang J , Chen M , et al. Recent advances in computer‐aided virtual screening and docking optimization for aptamer. Curr Top Med Chem. 2023;23(20):1985‐2000. doi:10.2174/1568026623666230623145802 PMID: 37357516. 37357516

[19] Farwa U , Raza MA . Heterocyclic compounds as a magic bullet for diabetes mellitus: a review. RSC Adv. 2022;12(35):22951‐22973. doi:10.1039/d2ra02697j 36105949 PMC9379558

[20] Bexell D , Svensson A , Bengzon J . Stem cell‐based therapy for malignant glioma. Cancer Treat Rev. 2013;39(4):358‐365. doi:10.1016/j.ctrv.2012.06.006 22795538

[21] Chen B , Zhou X , Yang L , et al. Glioma stem cell signature predicts the prognosis and the response to tumor treating fields treatment. CNS Neurosci Ther. 2022;28(12):2148‐2162. doi:10.1111/cns.13956 36070228 PMC9627385

[22] Sleire L , Forde H , Netland I , Leiss L , Skeie B , Enger P . Drug repurposing in cancer. Pharmacol Res. 2017;124:74‐91. doi:10.1016/j.phrs.2017.07.013 PMID: 28712971. 28712971

[23] Whibley C , Pharoah PD , Hollstein M . p53 polymorphisms: cancer implications. Nat Rev Cancer. 2009;9(2):95‐107. doi:10.1038/nrc2584 PMID: 19165225. 19165225

[24] Yang W , Stockwell B . Ferroptosis: death by lipid peroxidation. Trends Cell Biol. 2016;26(3):165‐176. doi:10.1016/j.tcb.2015.10.014 26653790 PMC4764384

[25] Xie Y , Hou W , Song Y , et al. Ferroptosis: process and function. Cell Death Differ. 2016;23(3):369‐379. doi:10.1038/cdd.2015.158 26794443 PMC5072448

[26] Lin S , Yang L , Yao Y , et al. Flubendazole demonstrates valid antitumor effects by inhibiting STAT3 and activating autophagy. J Exp Clin Cancer Res. 2019;38(1):293. doi:10.1186/s13046-019-1303-z 31287013 PMC6615228

[27] Zhang C , Liu X , Jin S , Chen Y , Guo R . Ferroptosis in cancer therapy: a novel approach to reversing drug resistance. Mol Cancer. 2022;21(1):47. doi:10.1186/s12943-022-01530-y 35151318 PMC8840702

