# Supplementary figures and images for: Repurposing flubendazole for glioblastoma ferroptosis by affecting xCT and TFRC proteins
Source: J Cell Mol Med. 2024 Nov 14;28(22):e70188. doi: 10.1111/jcmm.70188 (PMC11563996; doi:10.1111/jcmm.70188)

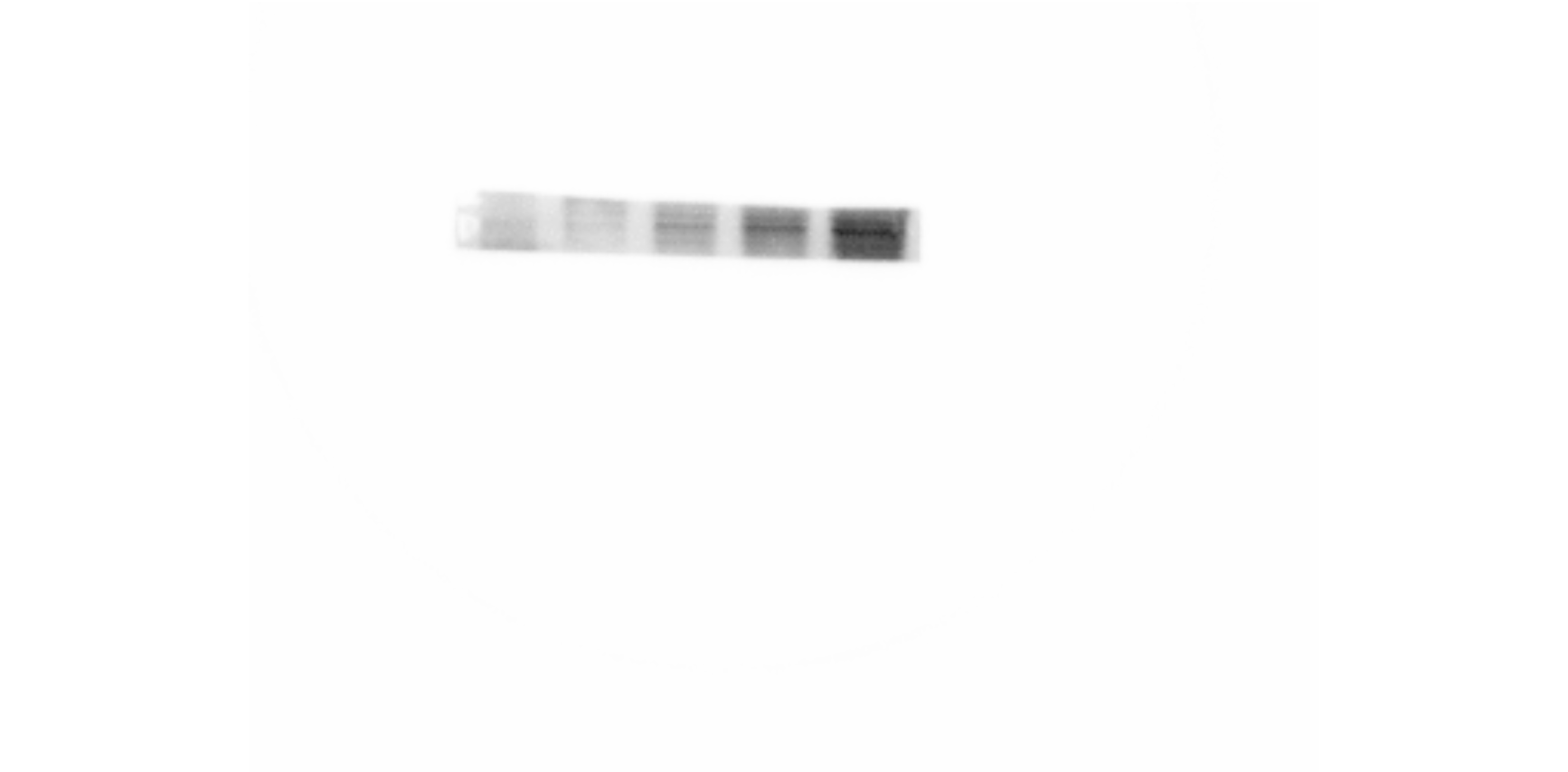

Supplement: Supplementary file 1 — Appendix S1: [file JCMM-28-e70188-s018.tif]

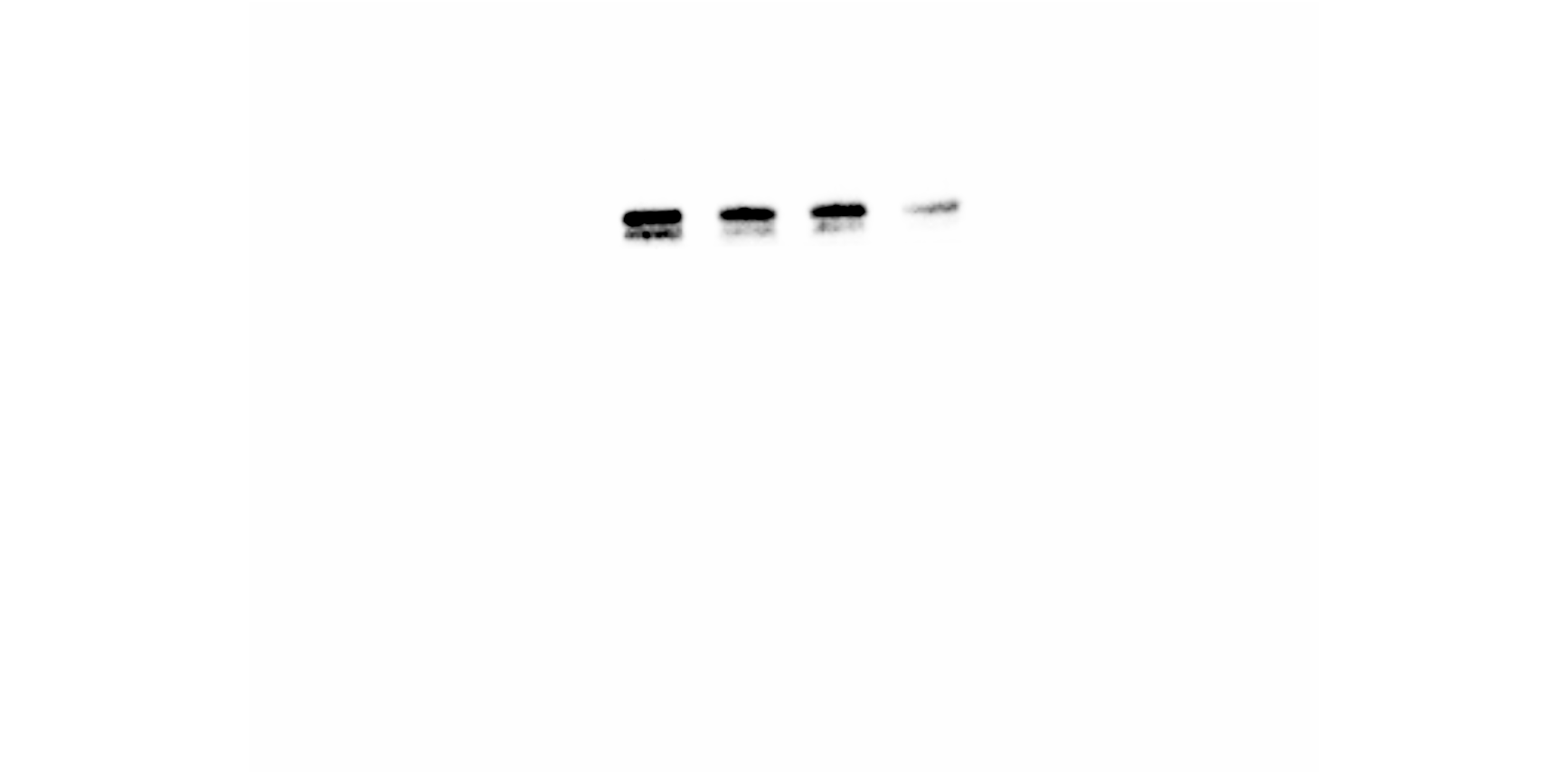

Supplement: Supplementary file 2 — Appendix S2: [file JCMM-28-e70188-s015.tif]

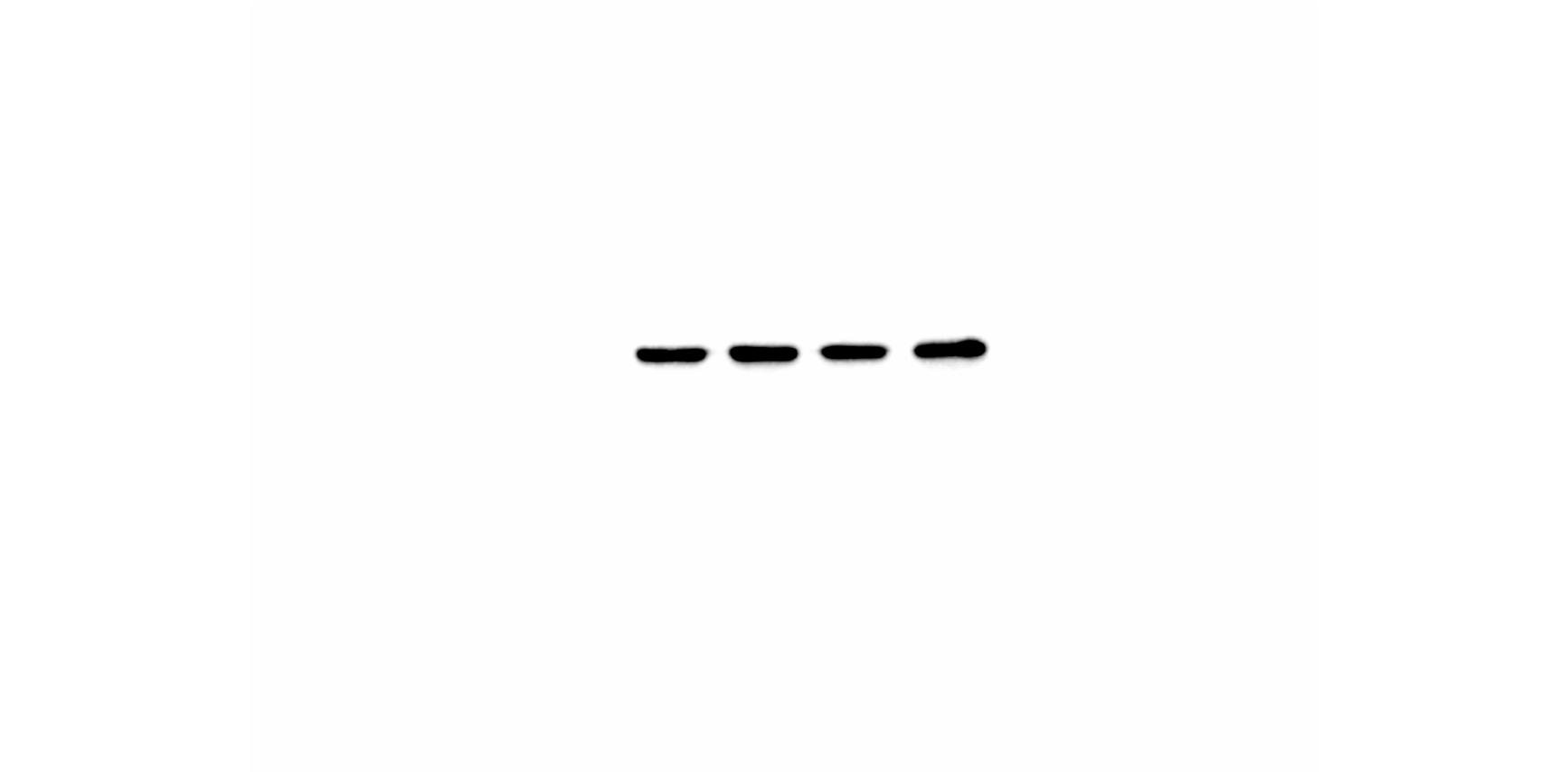

Supplement: Supplementary file 3 — Appendix S3: [file JCMM-28-e70188-s027.tif]

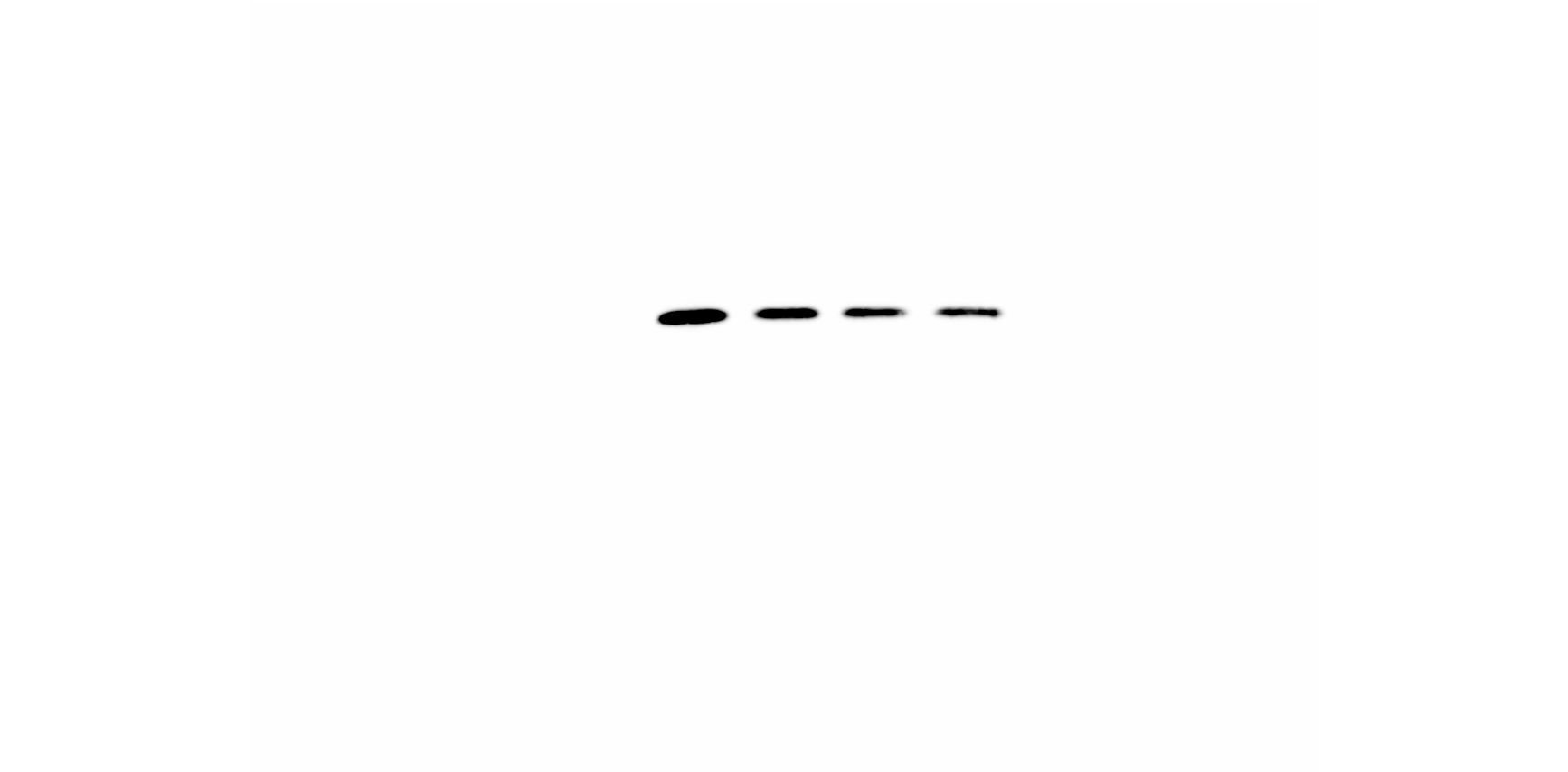

Supplement: Supplementary file 4 — Appendix S4: [file JCMM-28-e70188-s005.tif]

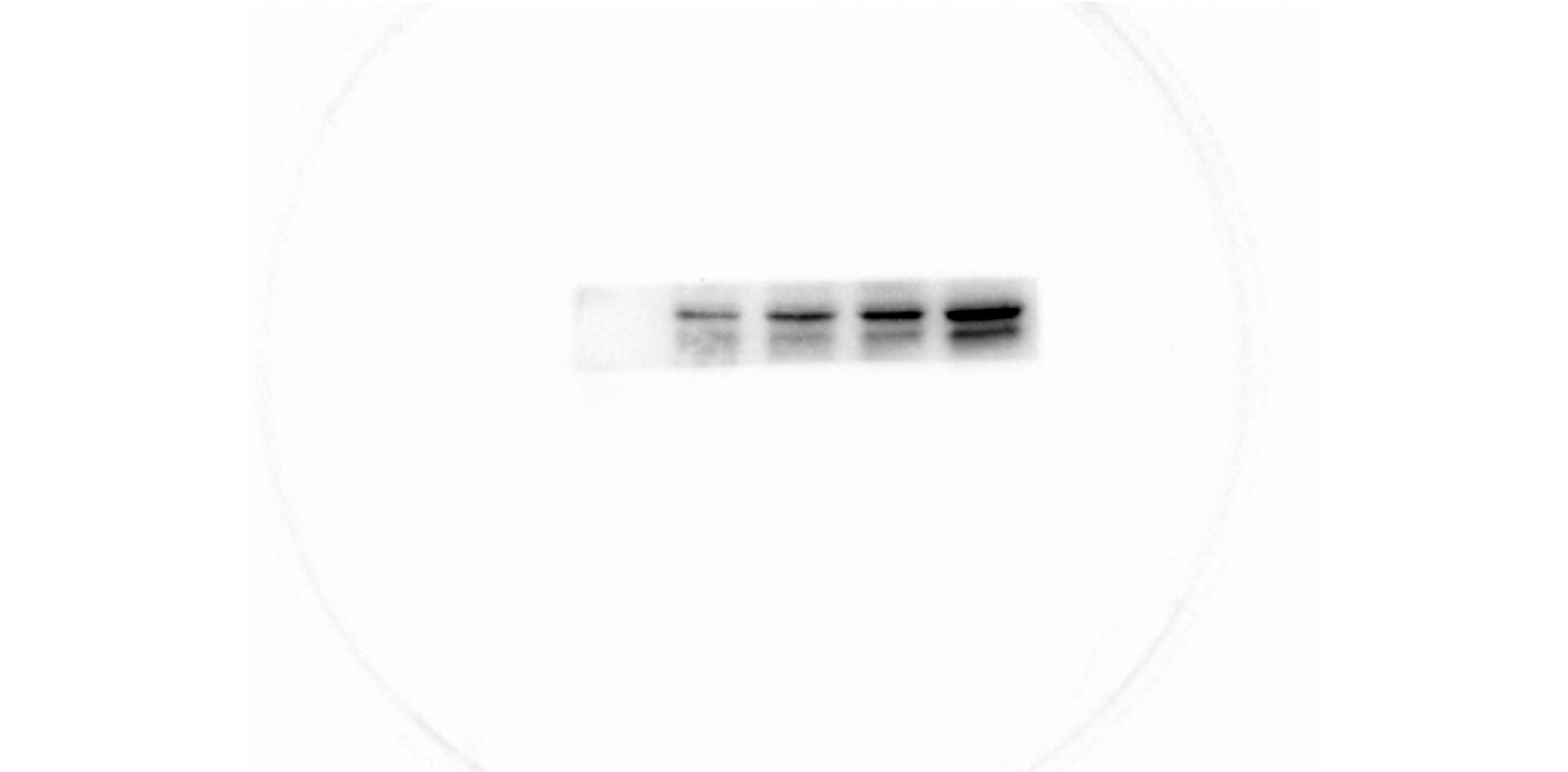

Supplement: Supplementary file 5 — Appendix S5: [file JCMM-28-e70188-s022.tif]

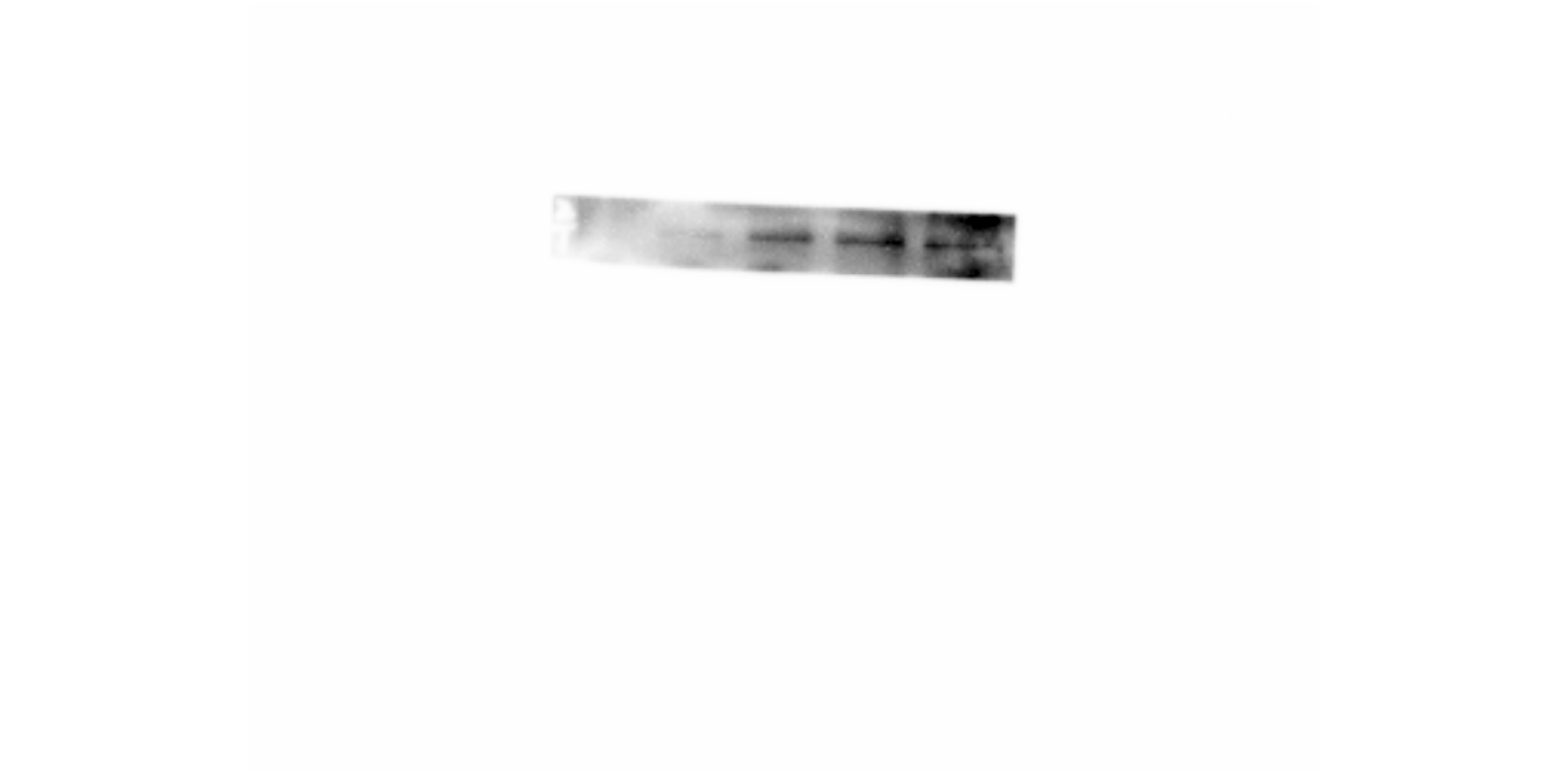

Supplement: Supplementary file 6 — Appendix S6: [file JCMM-28-e70188-s009.tif]

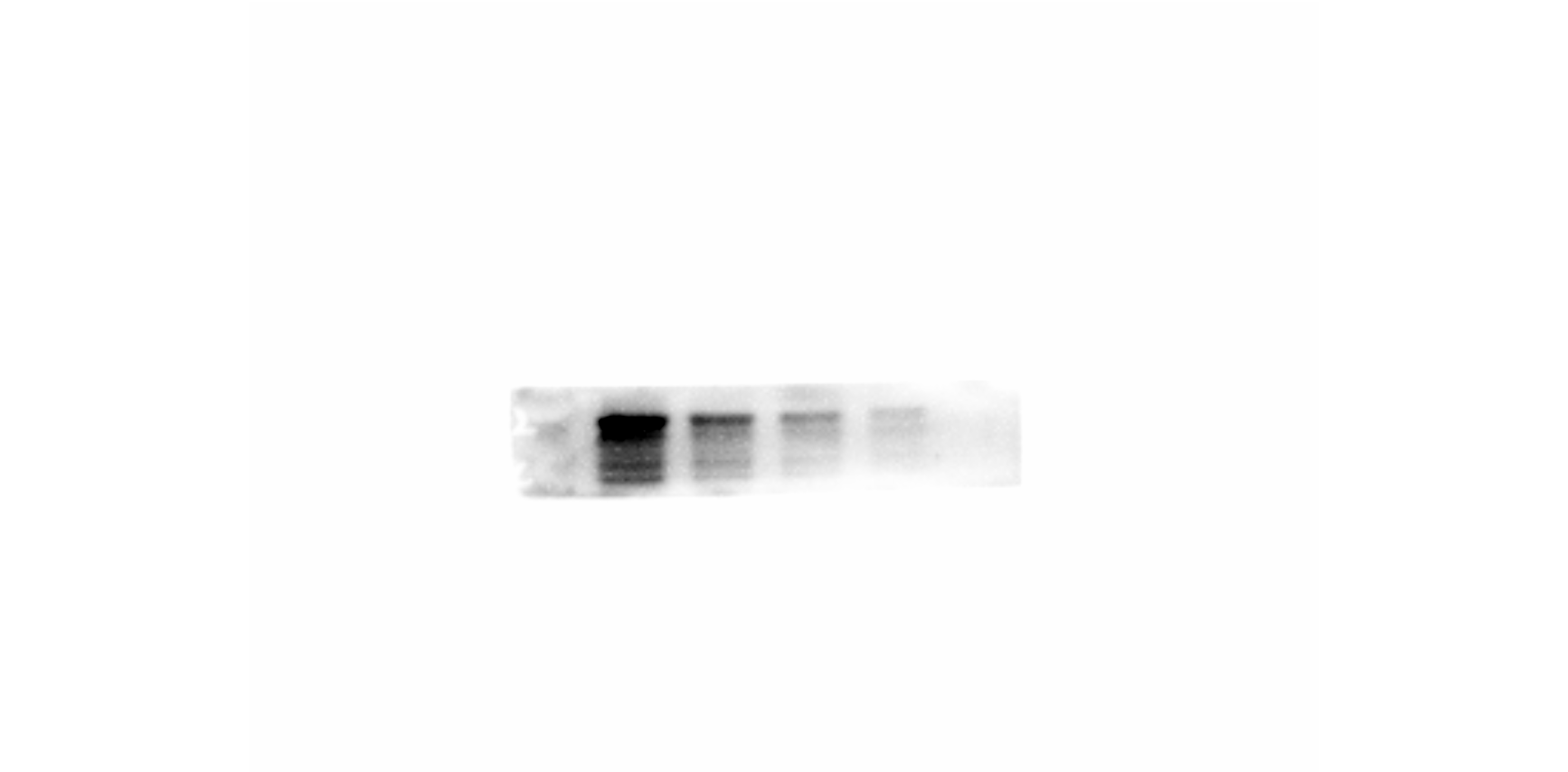

Supplement: Supplementary file 7 — Appendix S7: [file JCMM-28-e70188-s024.tif]

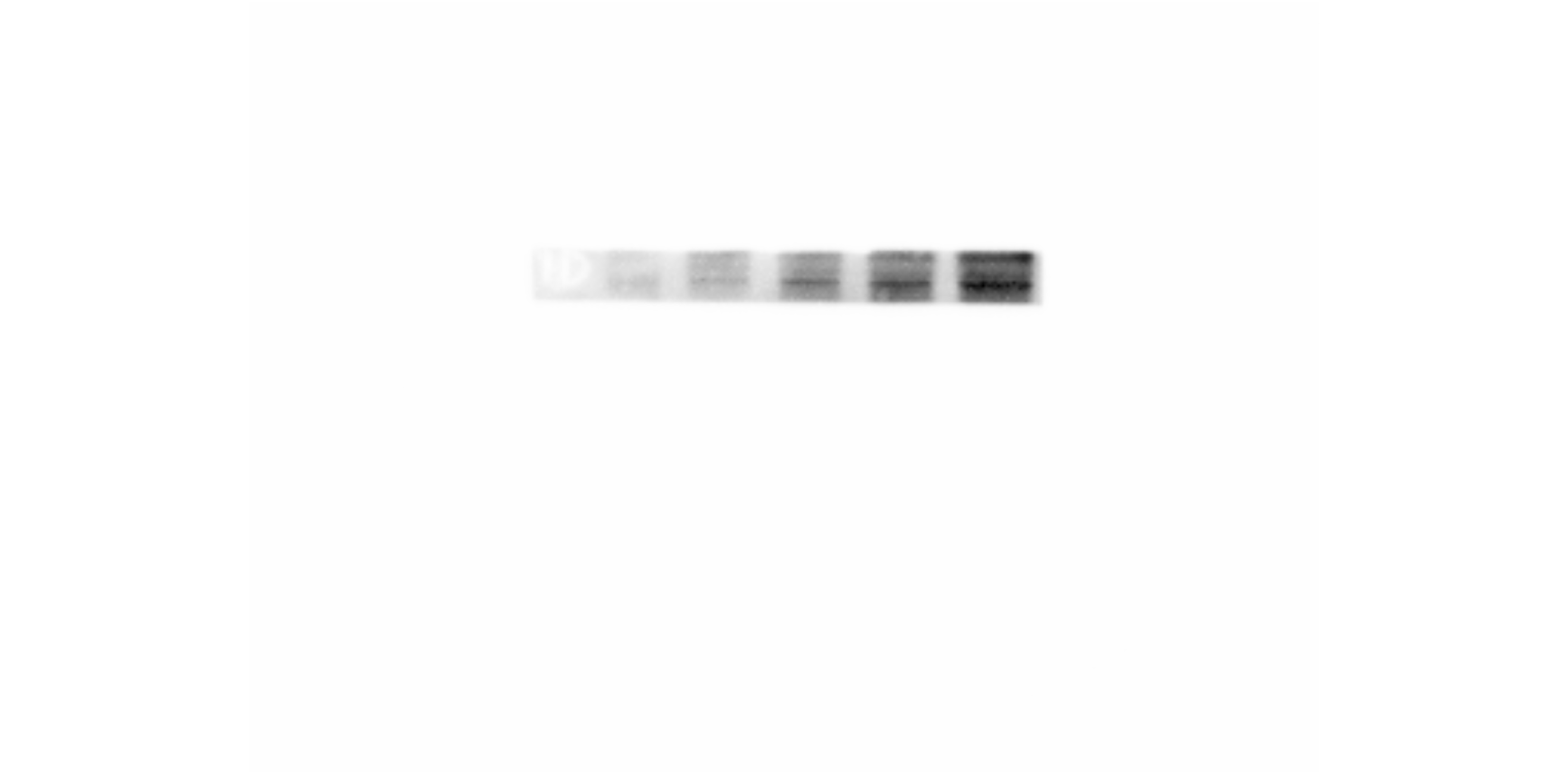

Supplement: Supplementary file 8 — Appendix S8: [file JCMM-28-e70188-s023.tif]

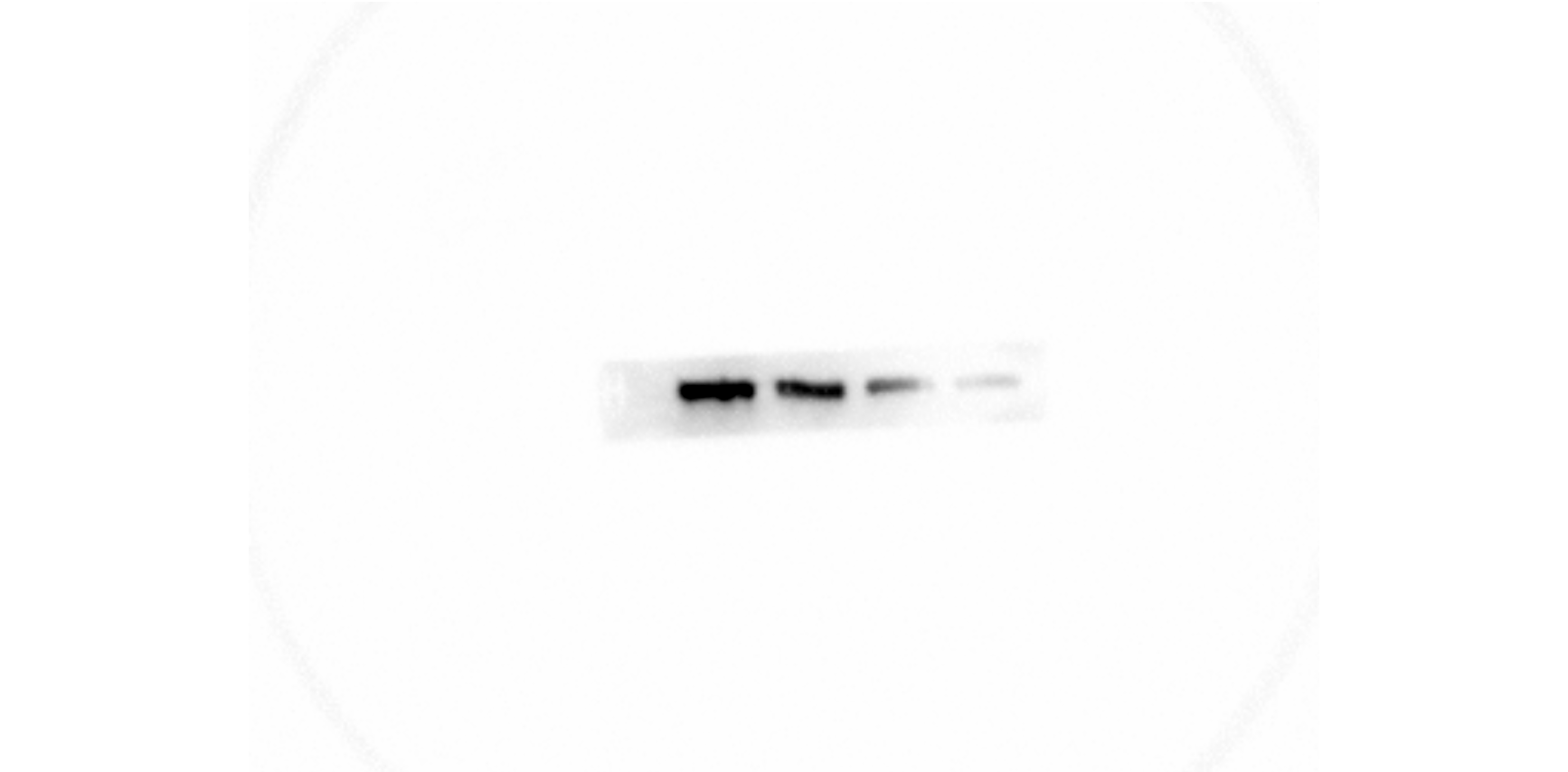

Supplement: Supplementary file 9 — Appendix S9: [file JCMM-28-e70188-s011.tif]

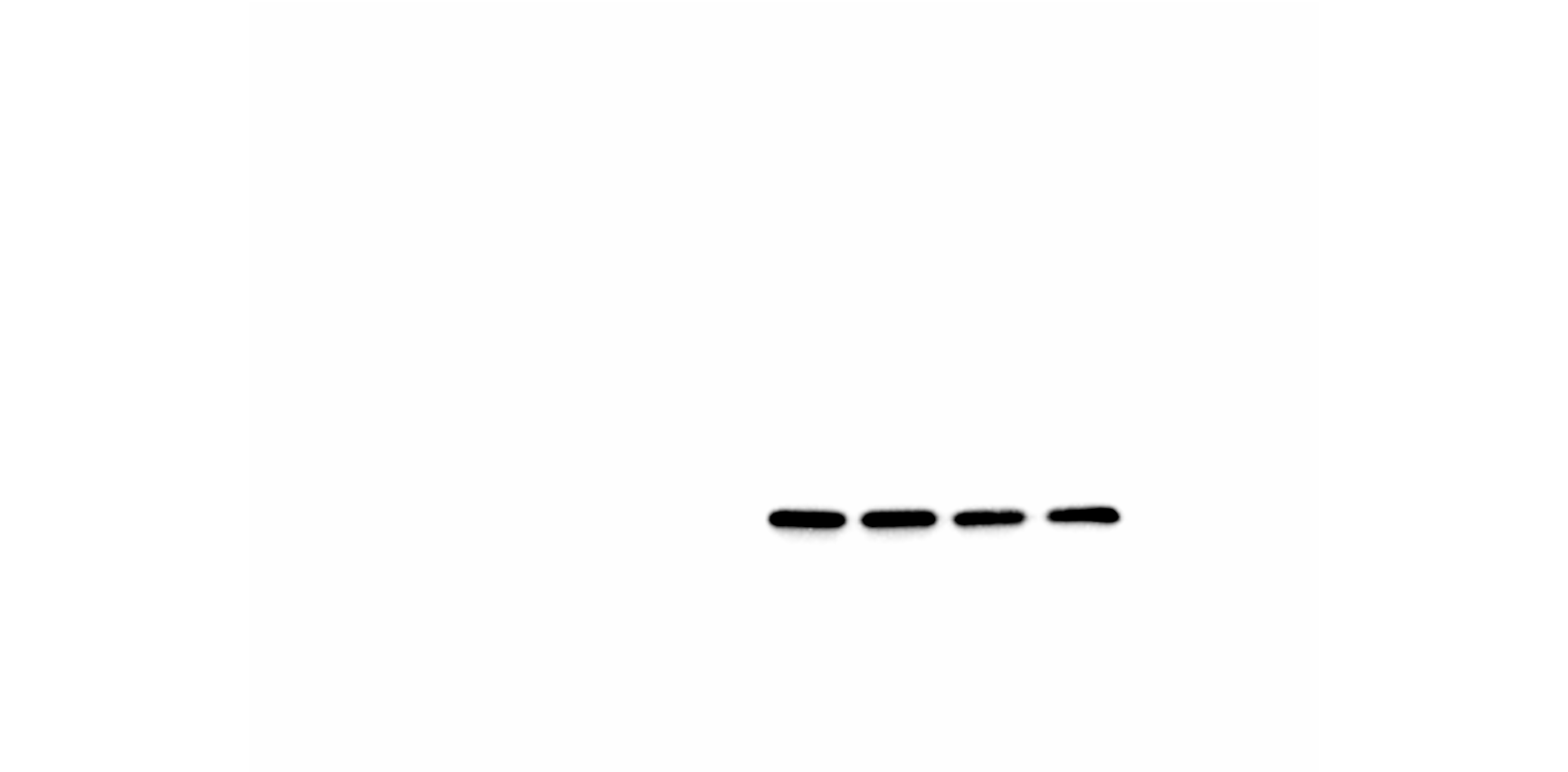

Supplement: Supplementary file 10 — Appendix S10. [file JCMM-28-e70188-s026.tif]

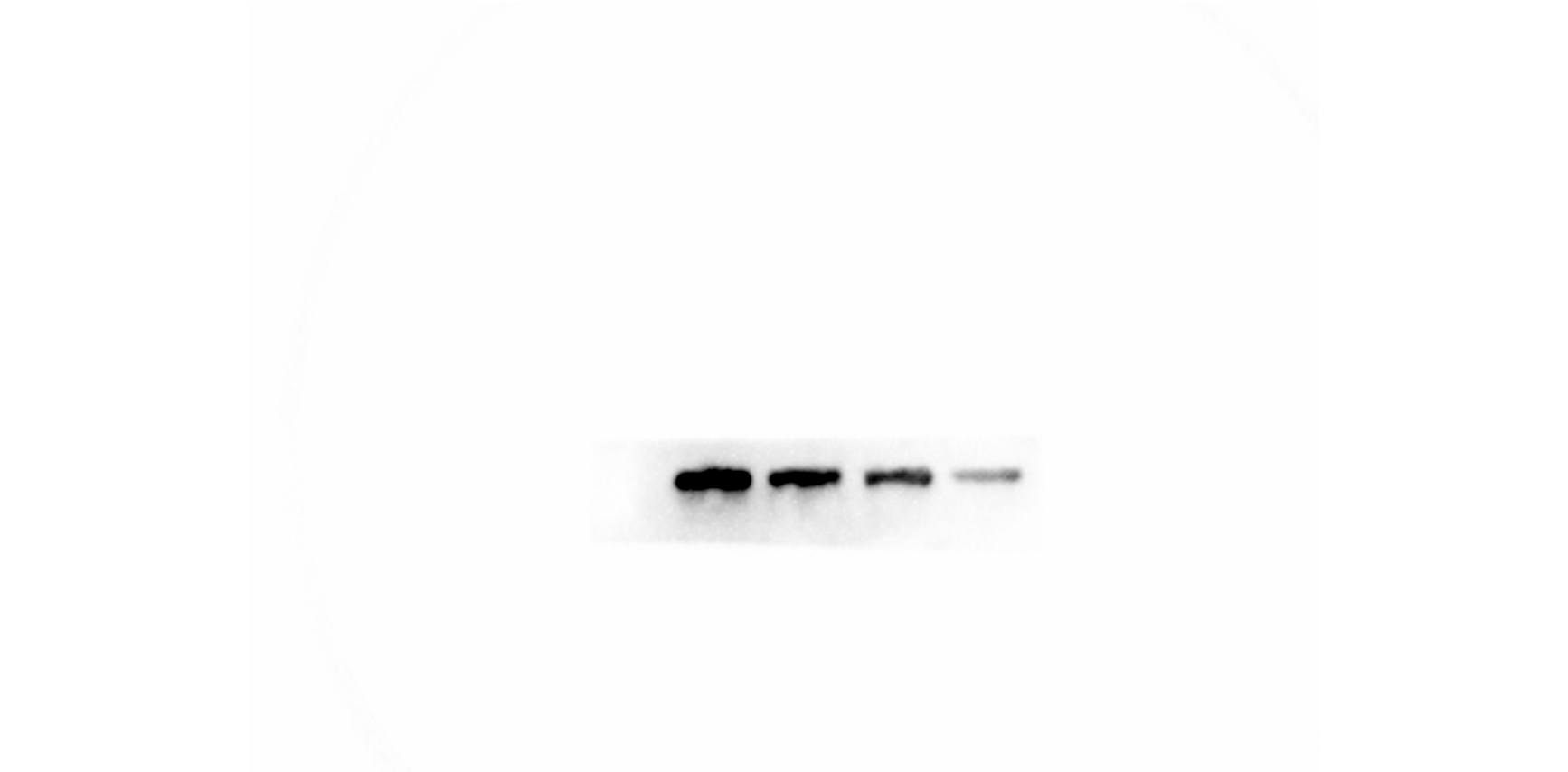

Supplement: Supplementary file 11 — Appendix S11. [file JCMM-28-e70188-s012.tif]

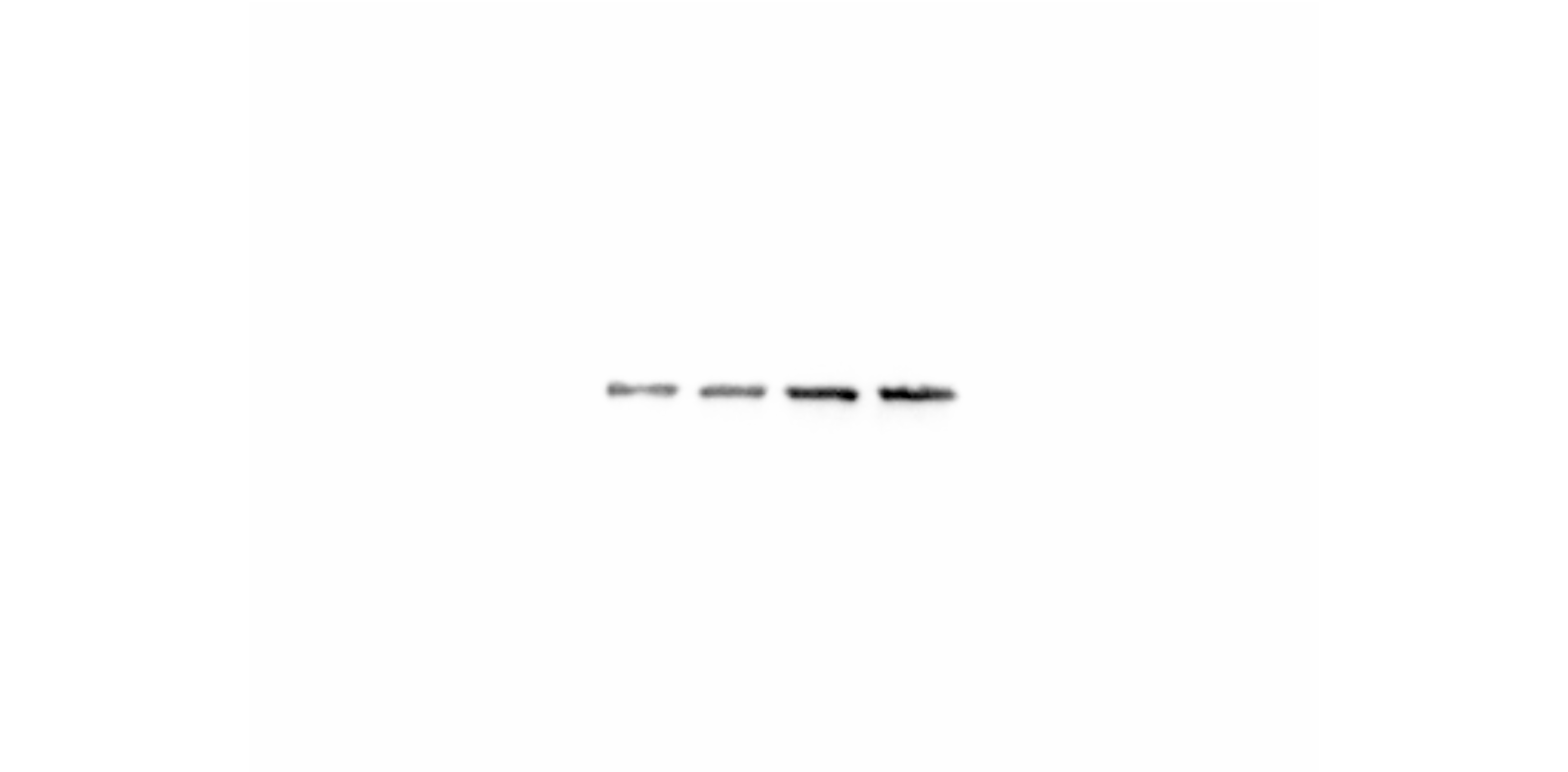

Supplement: Supplementary file 12 — Appendix S12. [file JCMM-28-e70188-s014.tif]

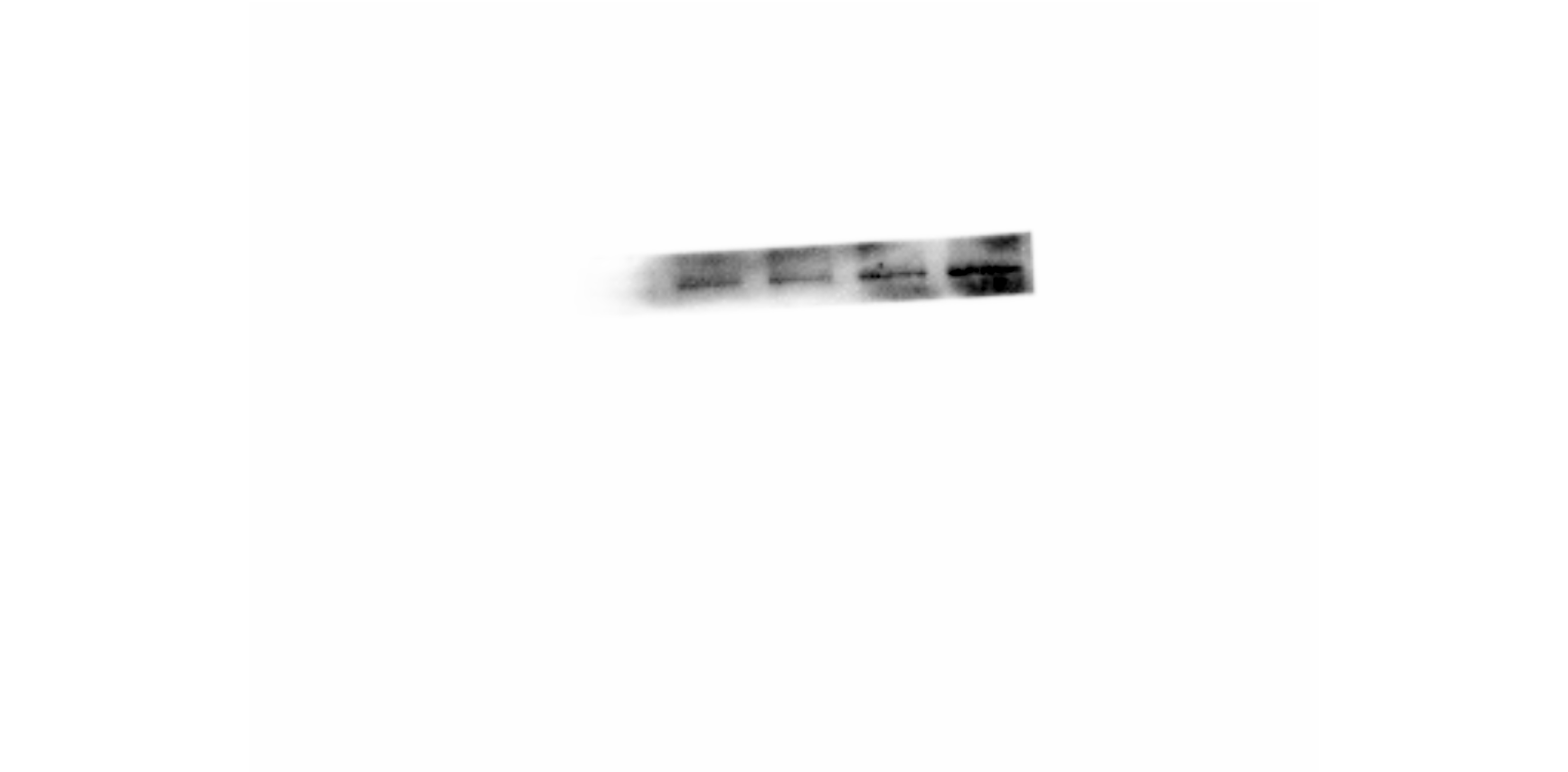

Supplement: Supplementary file 13 — Appendix S13. [file JCMM-28-e70188-s001.tif]

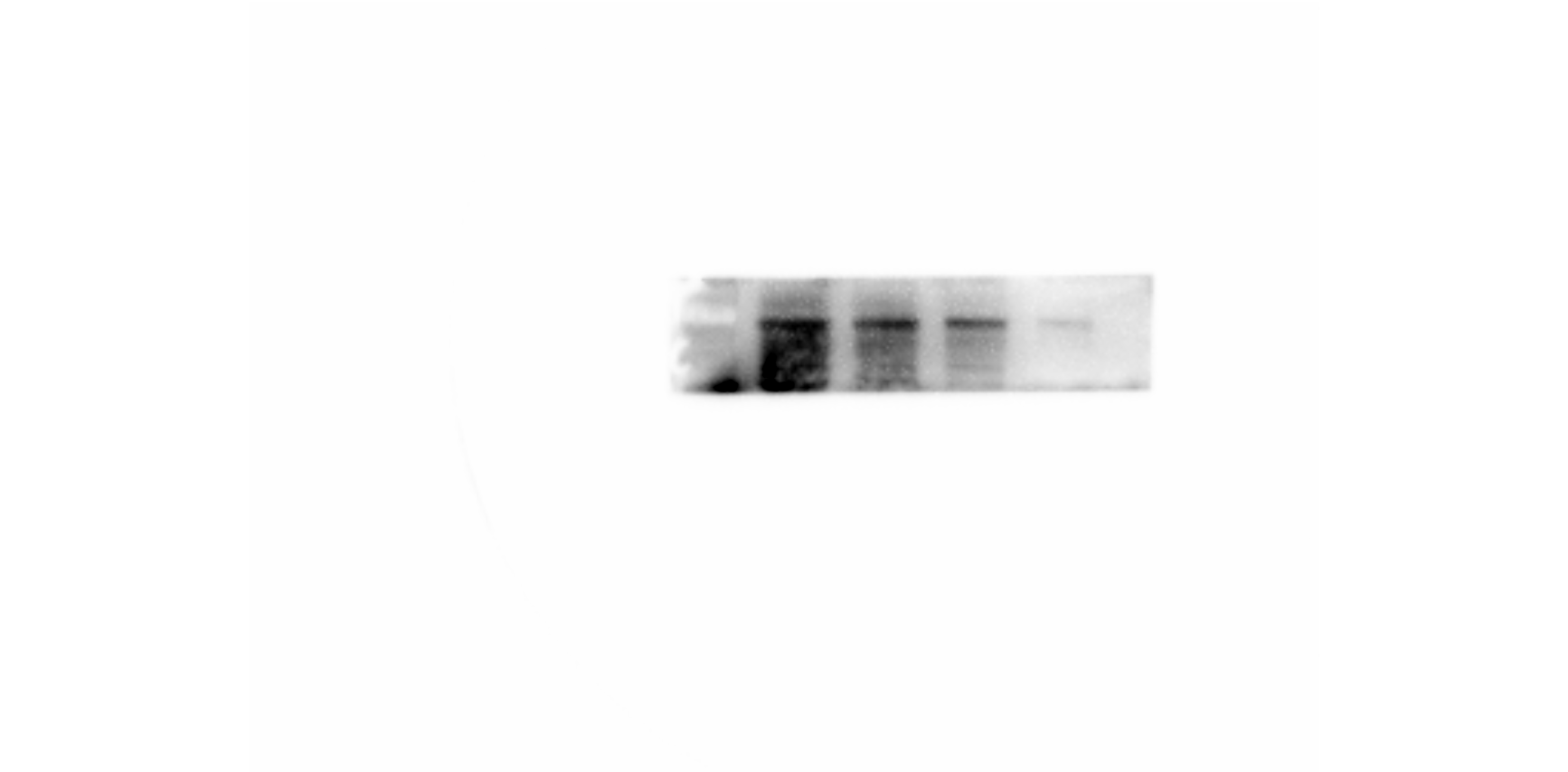

Supplement: Supplementary file 14 — Appendix S14. [file JCMM-28-e70188-s010.tif]

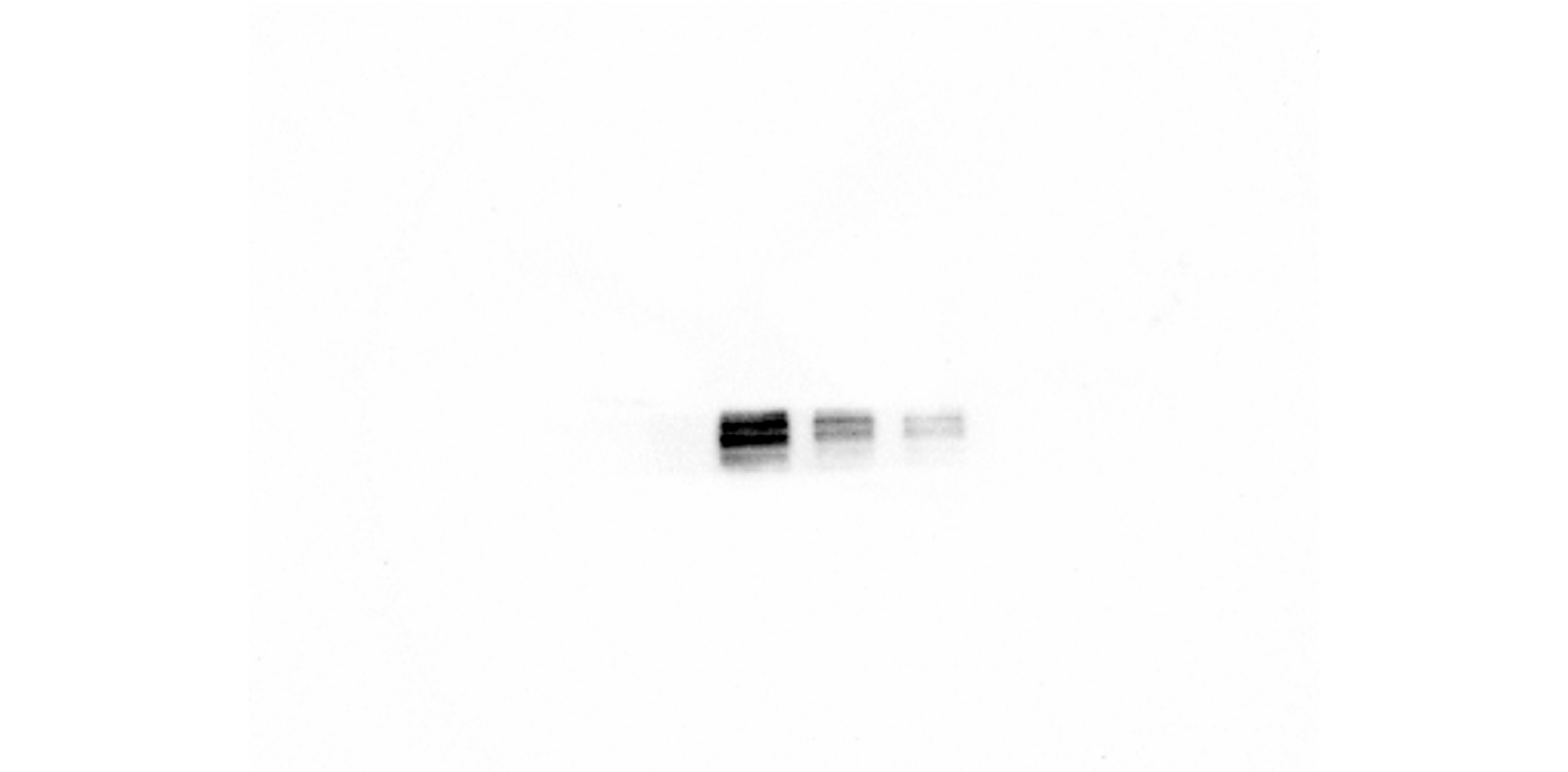

Supplement: Supplementary file 15 — Appendix S15. [file JCMM-28-e70188-s025.tif]

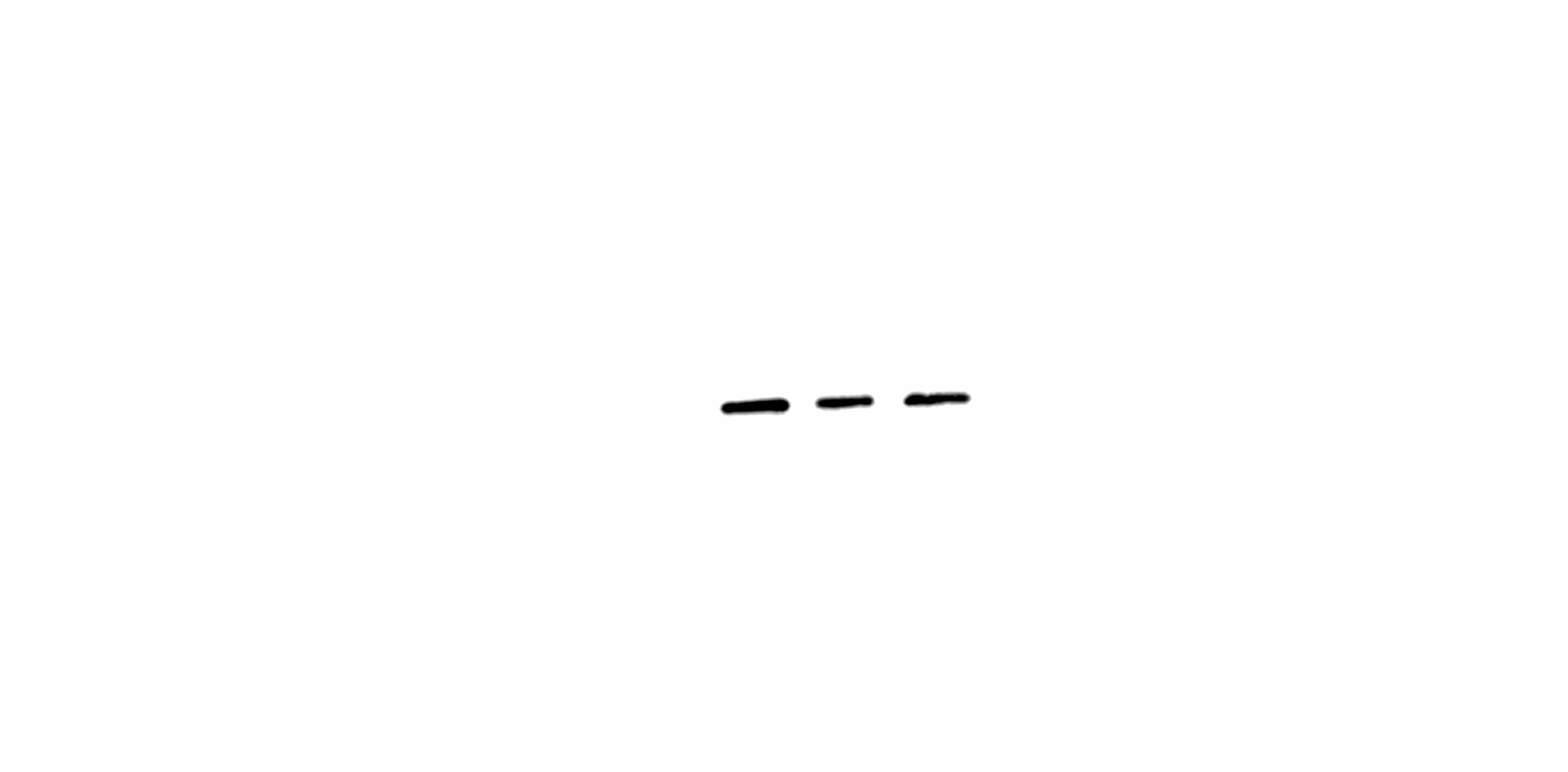

Supplement: Supplementary file 16 — Appendix S16. [file JCMM-28-e70188-s028.tif]

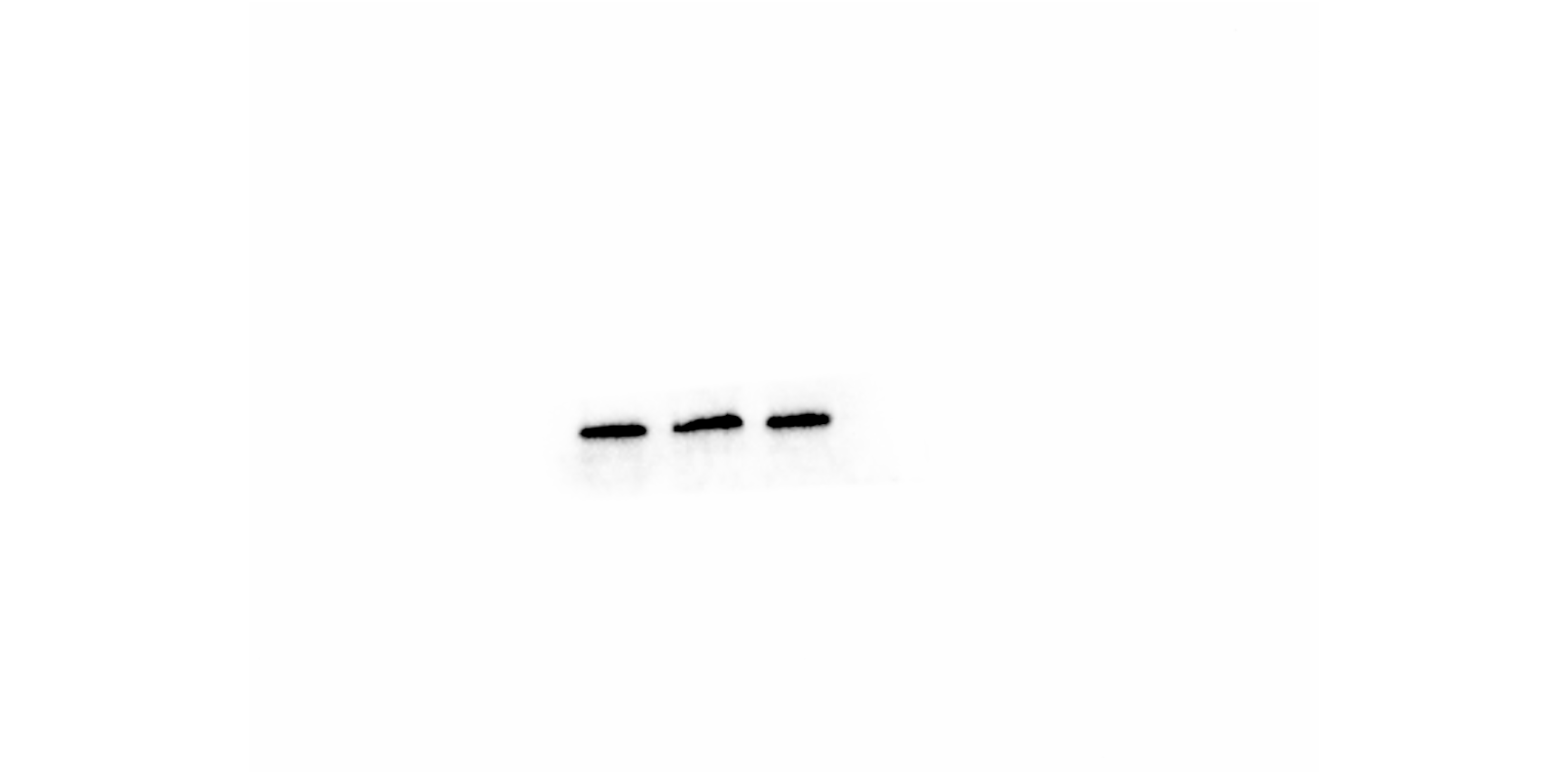

Supplement: Supplementary file 17 — Appendix S17. [file JCMM-28-e70188-s008.tif]

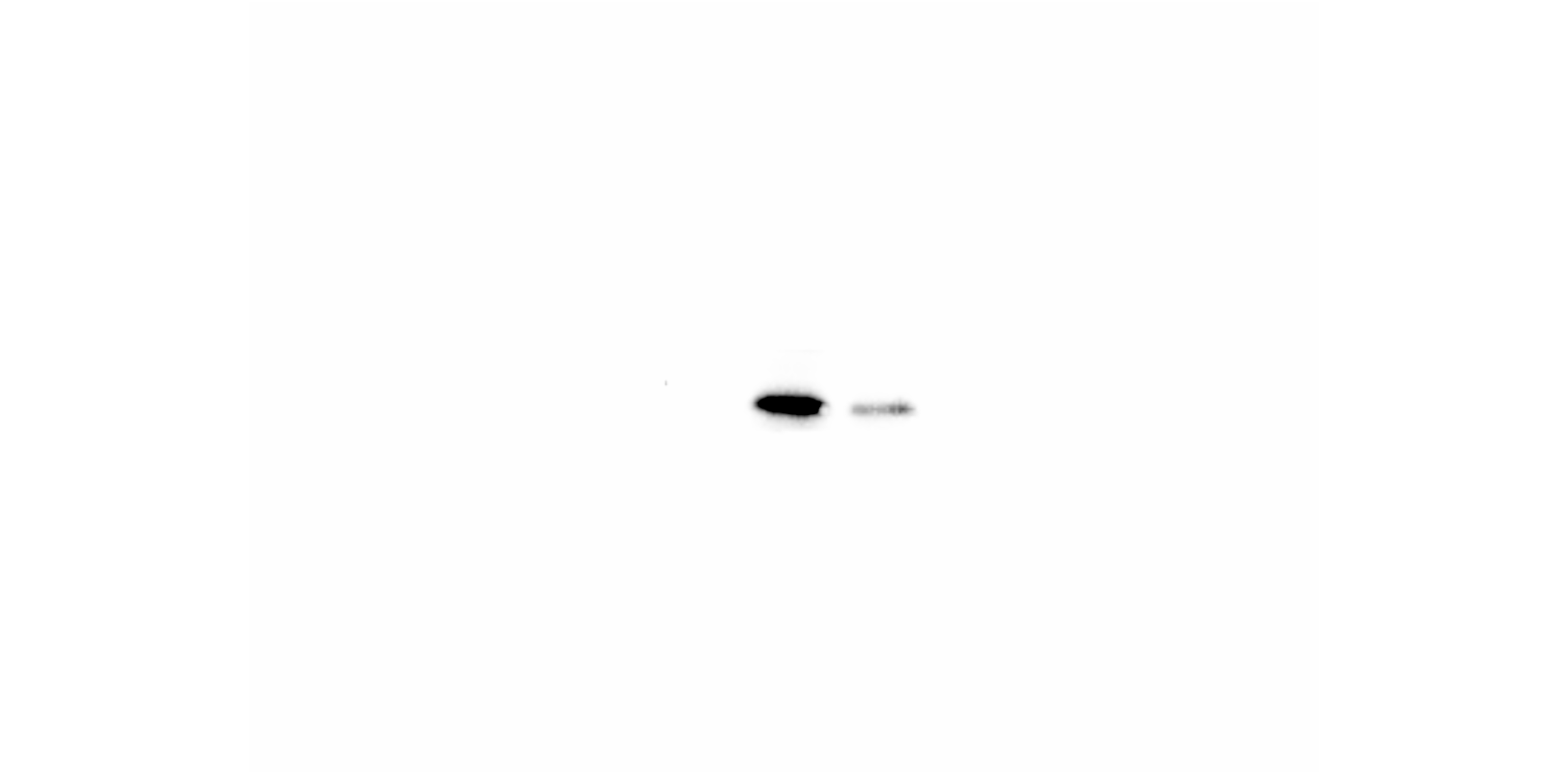

Supplement: Supplementary file 18 — Appendix S18. [file JCMM-28-e70188-s017.tif]

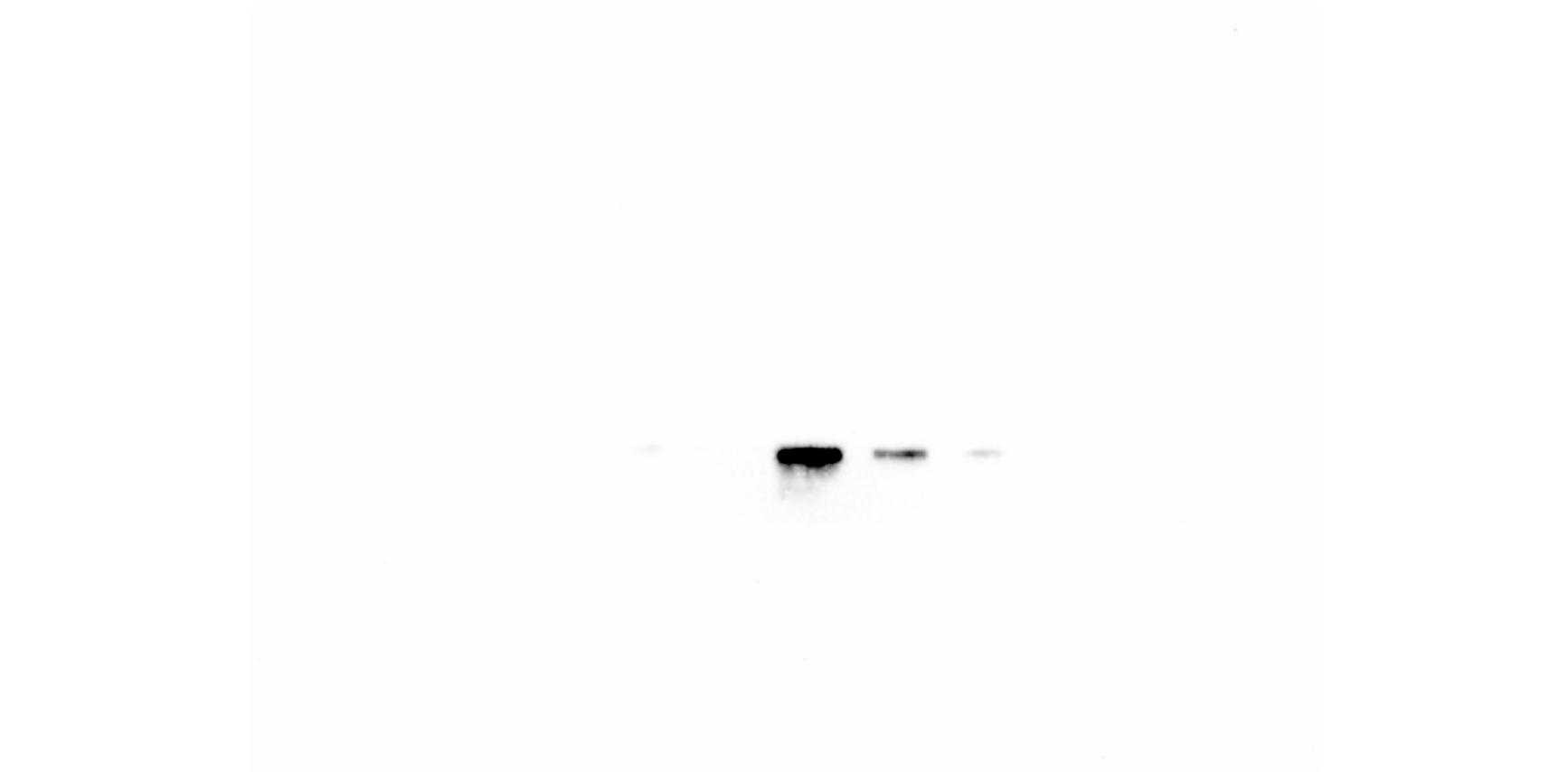

Supplement: Supplementary file 19 — Appendix S19. [file JCMM-28-e70188-s006.tif]

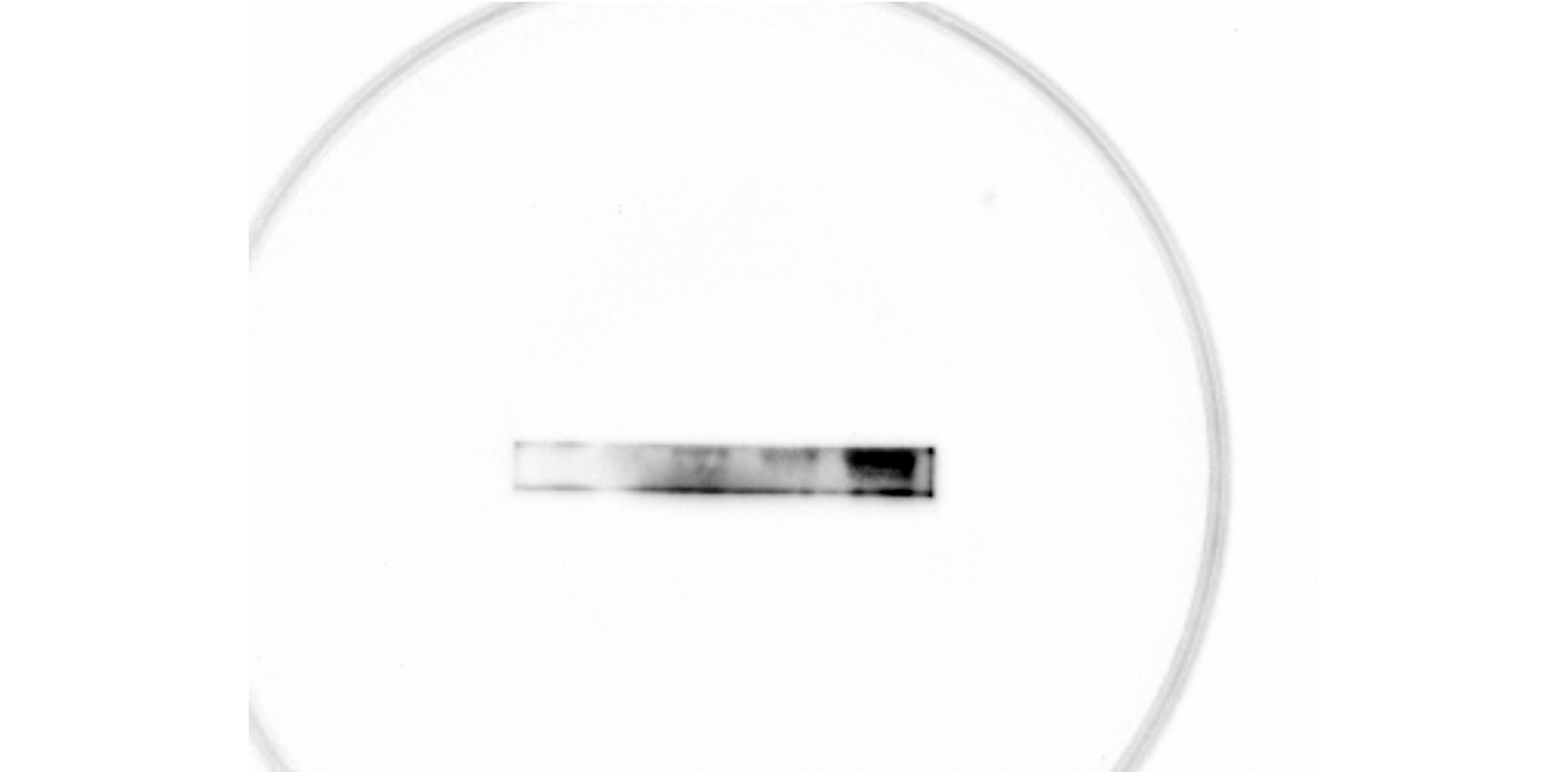

Supplement: Supplementary file 20 — Appendix S20. [file JCMM-28-e70188-s007.tif]

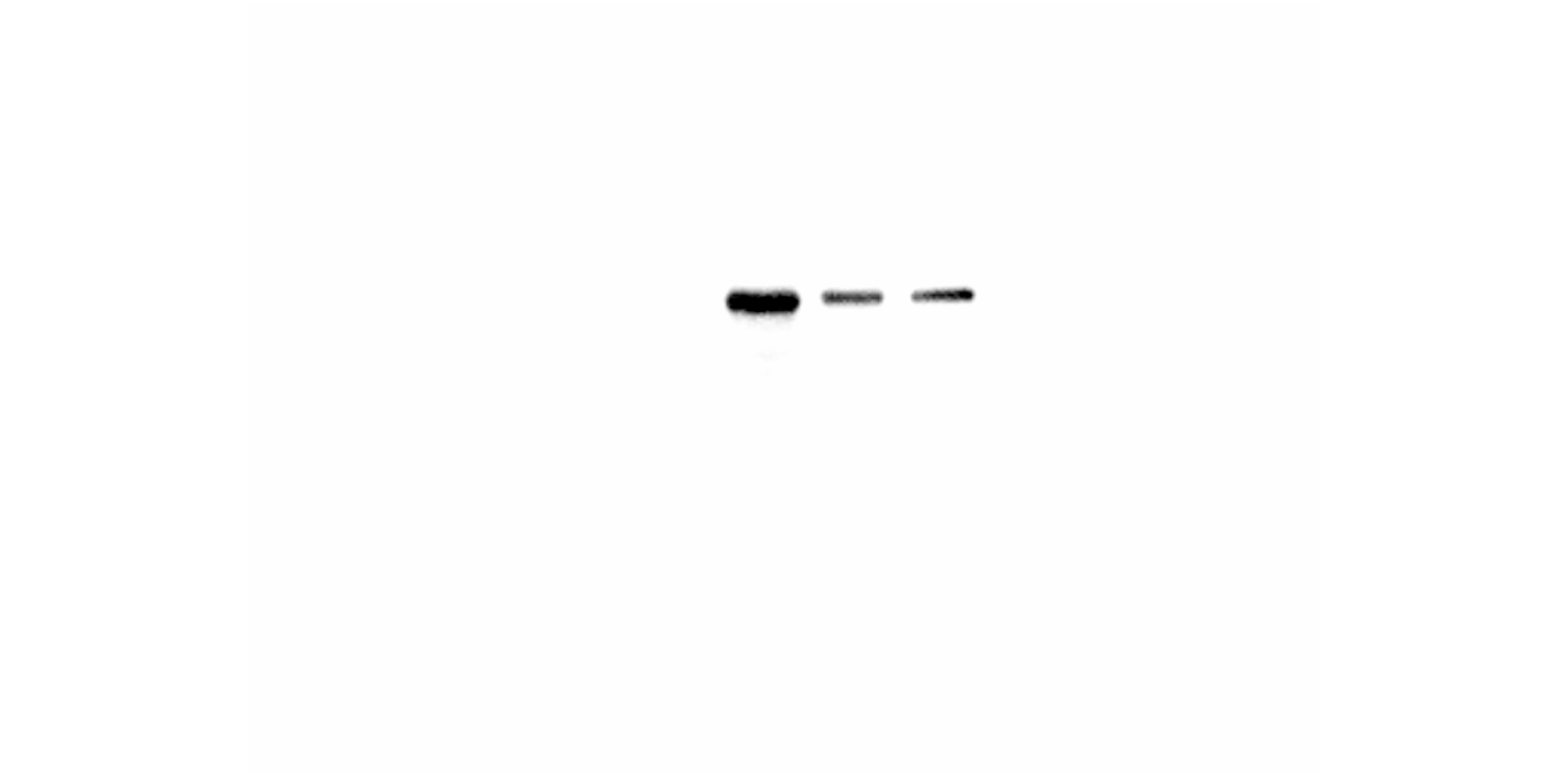

Supplement: Supplementary file 21 — Appendix S21. [file JCMM-28-e70188-s016.tif]

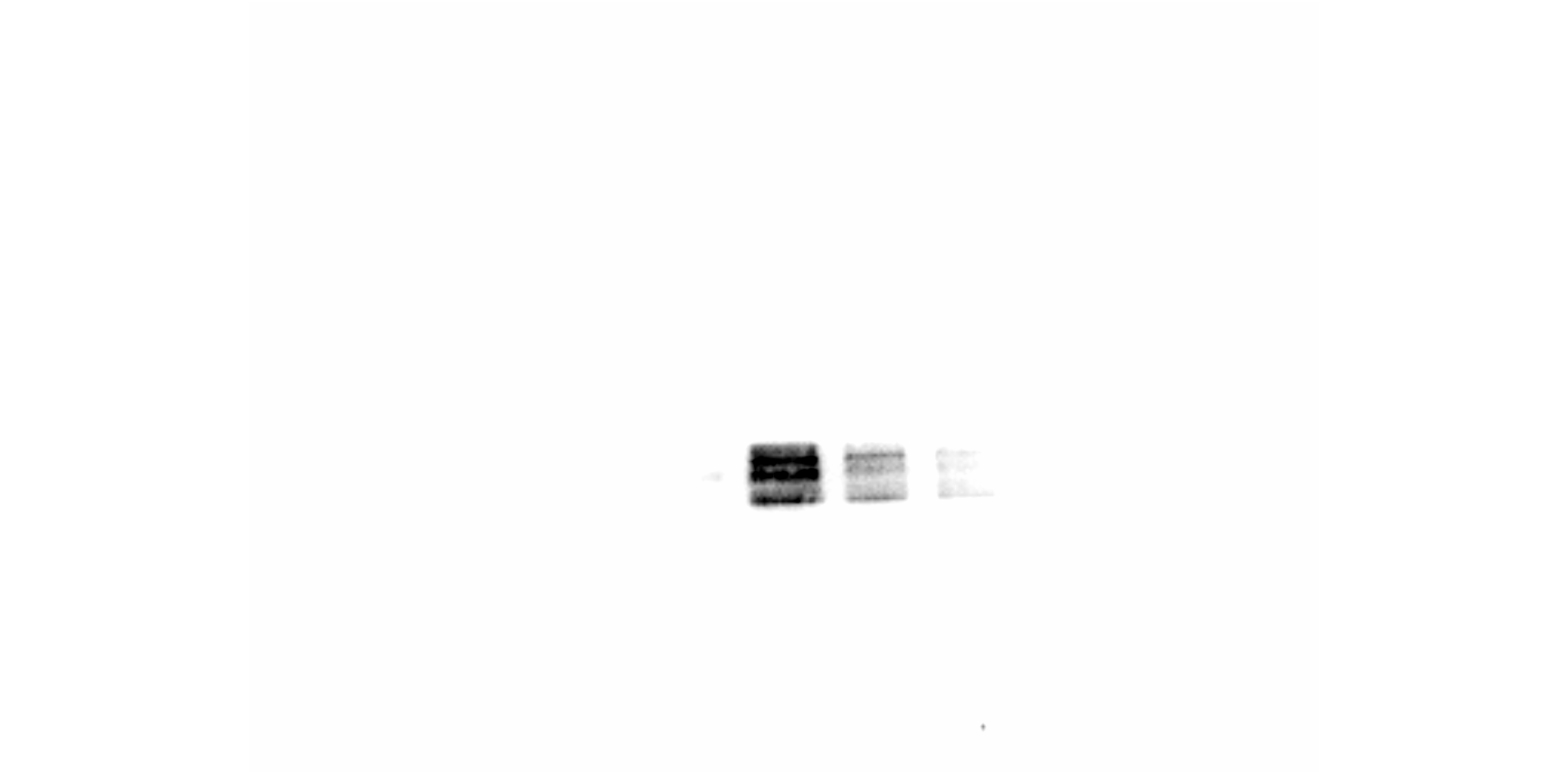

Supplement: Supplementary file 22 — Appendix S22. [file JCMM-28-e70188-s019.tif]

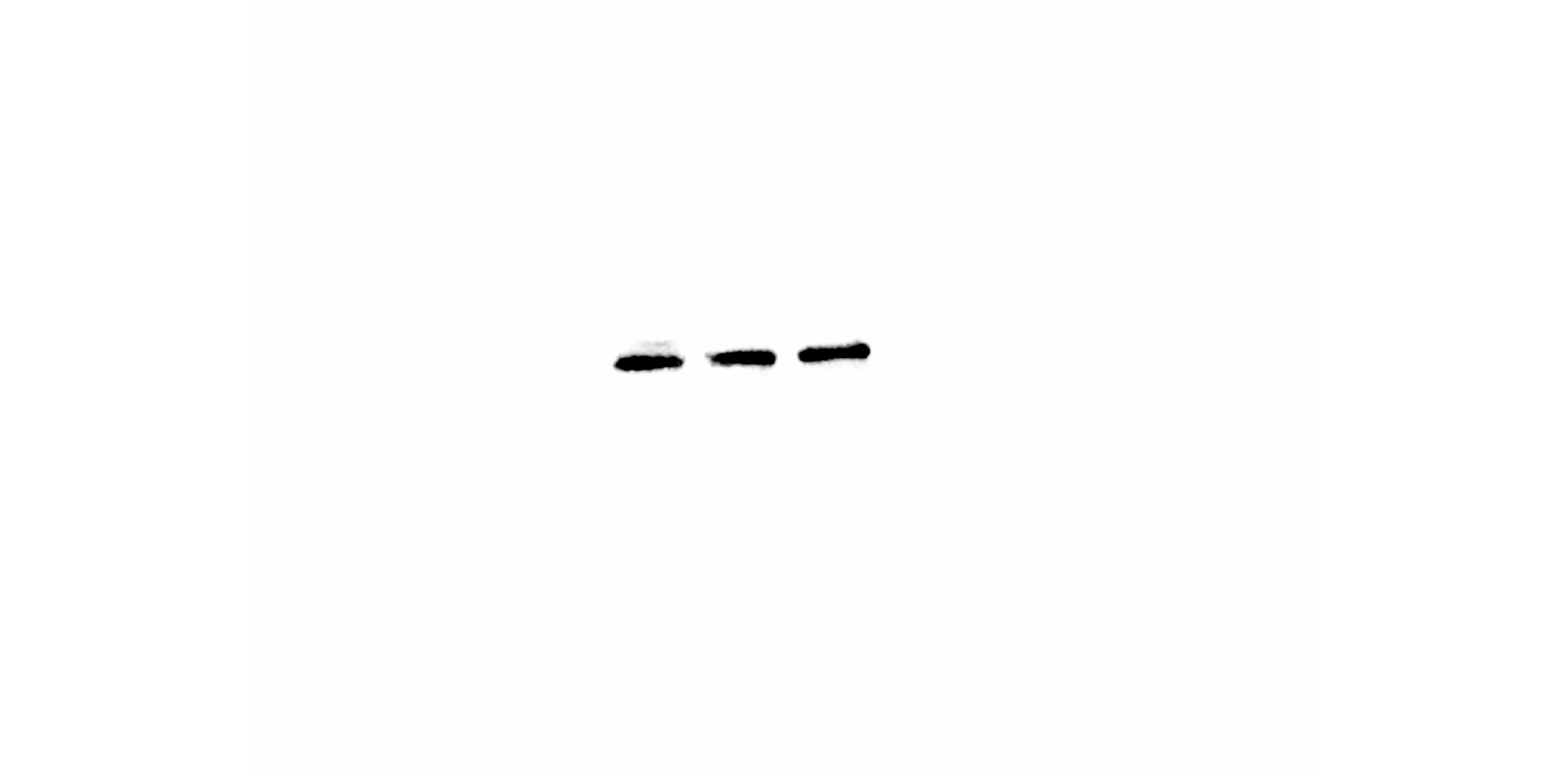

Supplement: Supplementary file 23 — Appendix S23. [file JCMM-28-e70188-s004.tif]

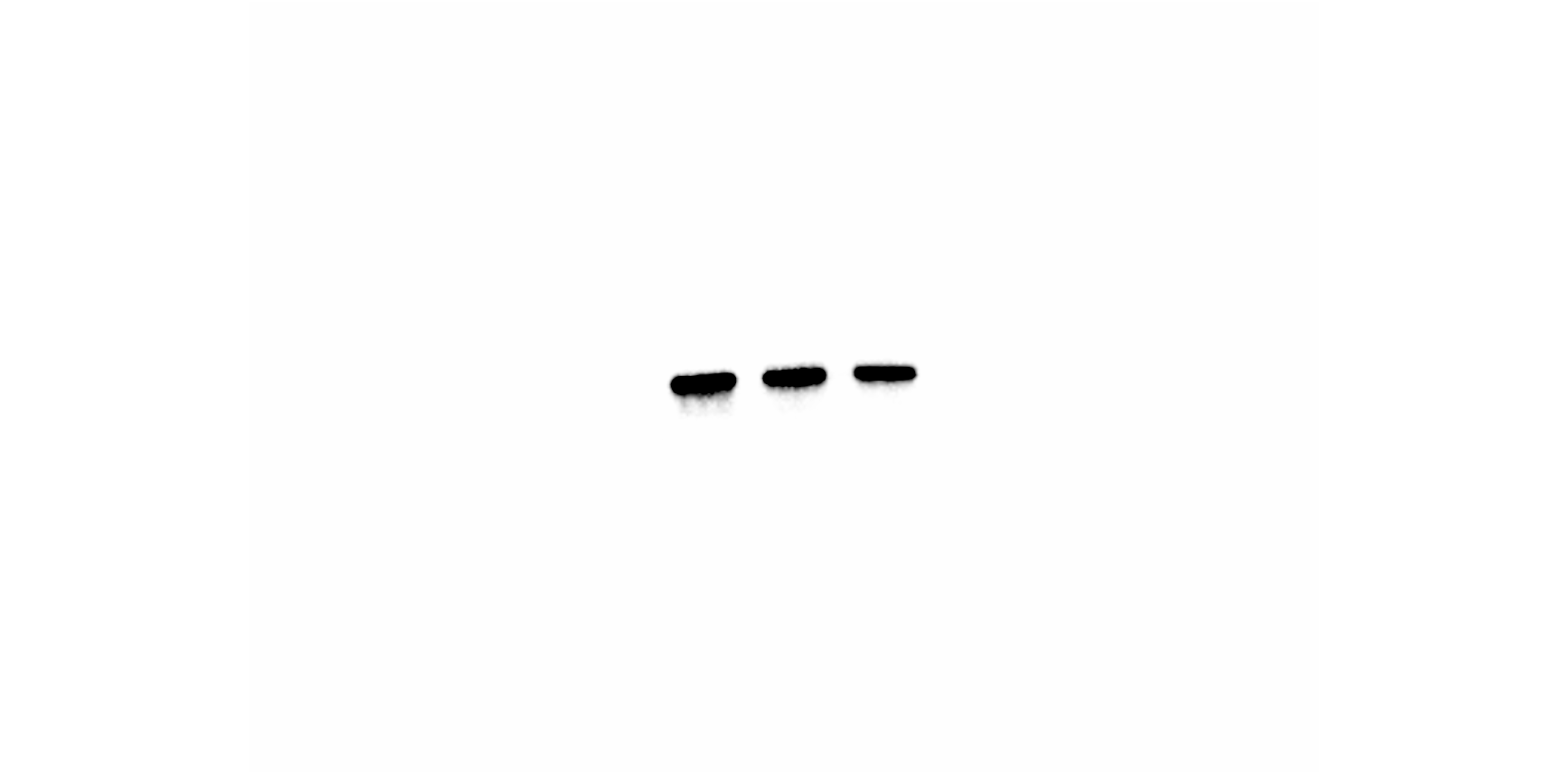

Supplement: Supplementary file 24 — Appendix S24. [file JCMM-28-e70188-s021.tif]

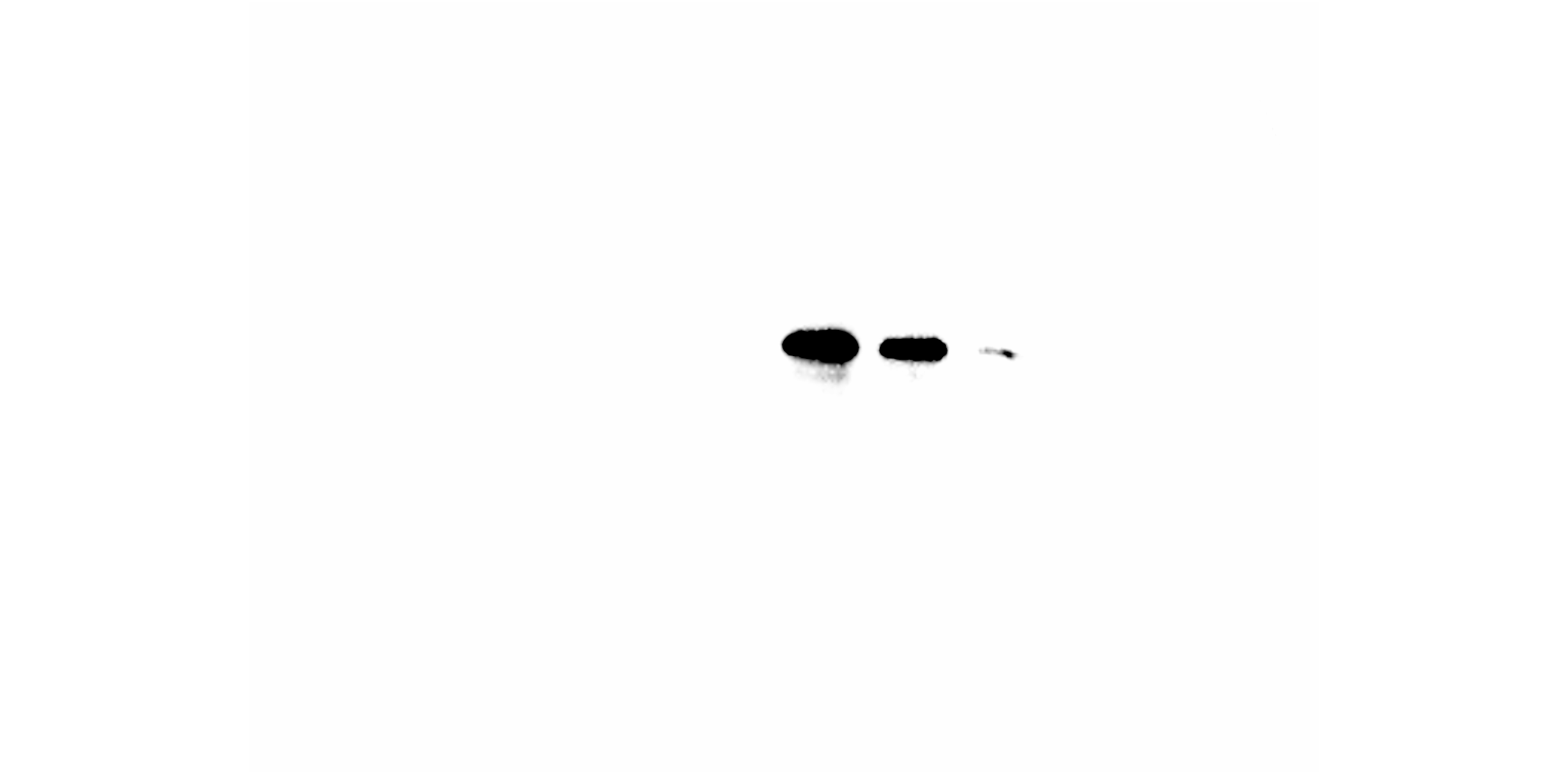

Supplement: Supplementary file 25 — Appendix S25. [file JCMM-28-e70188-s002.tif]

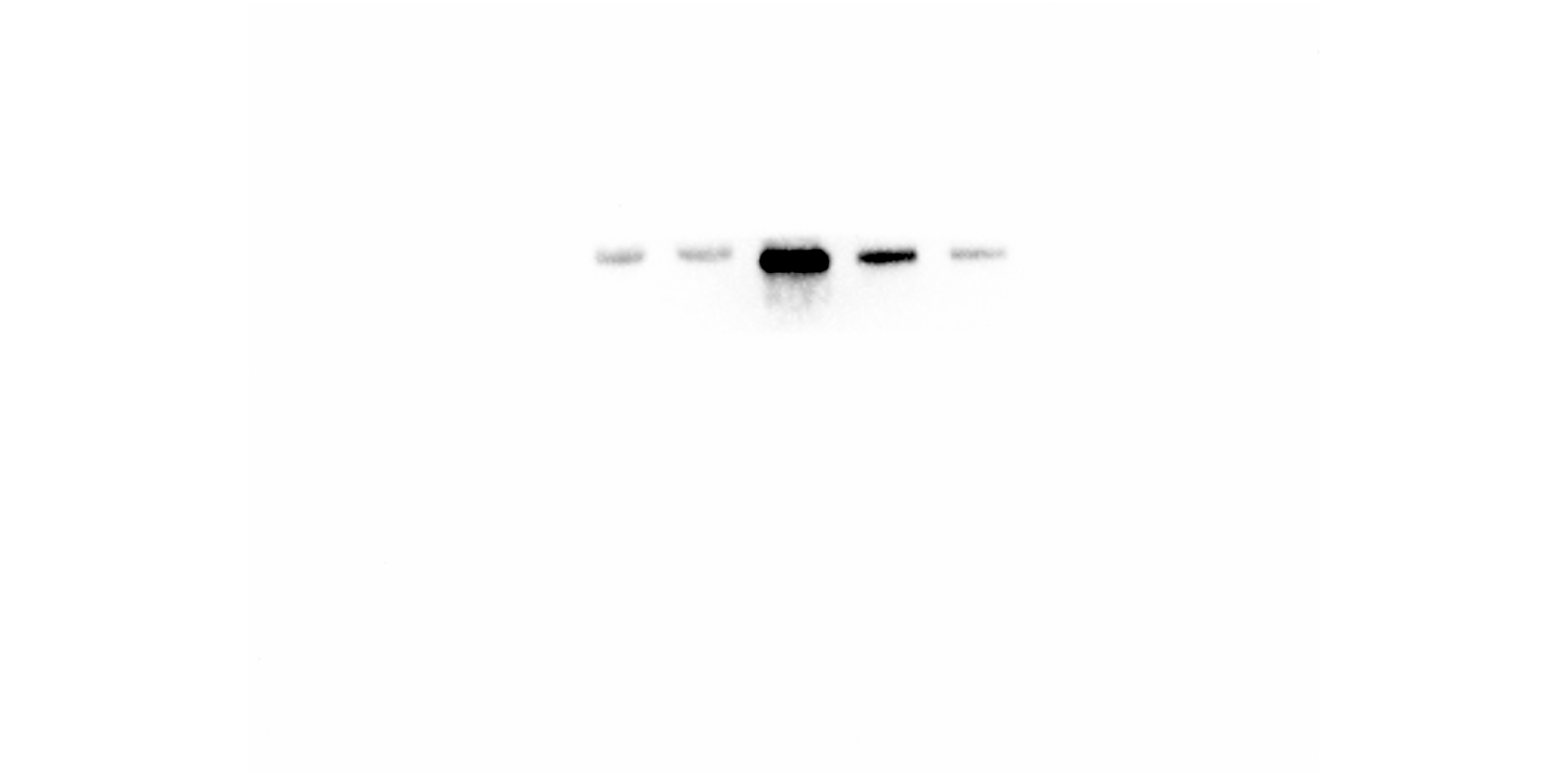

Supplement: Supplementary file 26 — Appendix S26. [file JCMM-28-e70188-s003.tif]

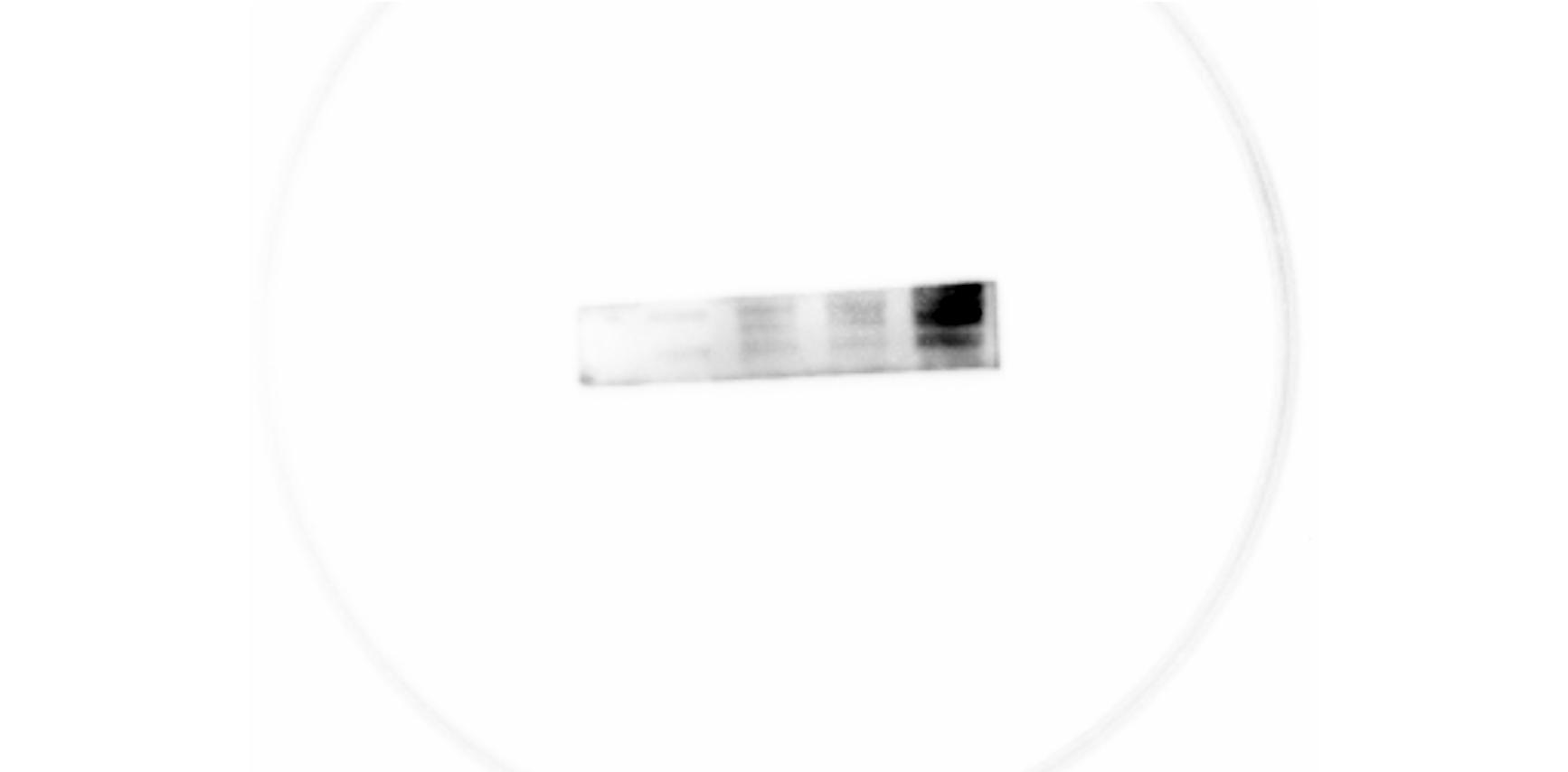

Supplement: Supplementary file 27 — Appendix S27. [file JCMM-28-e70188-s020.tif]

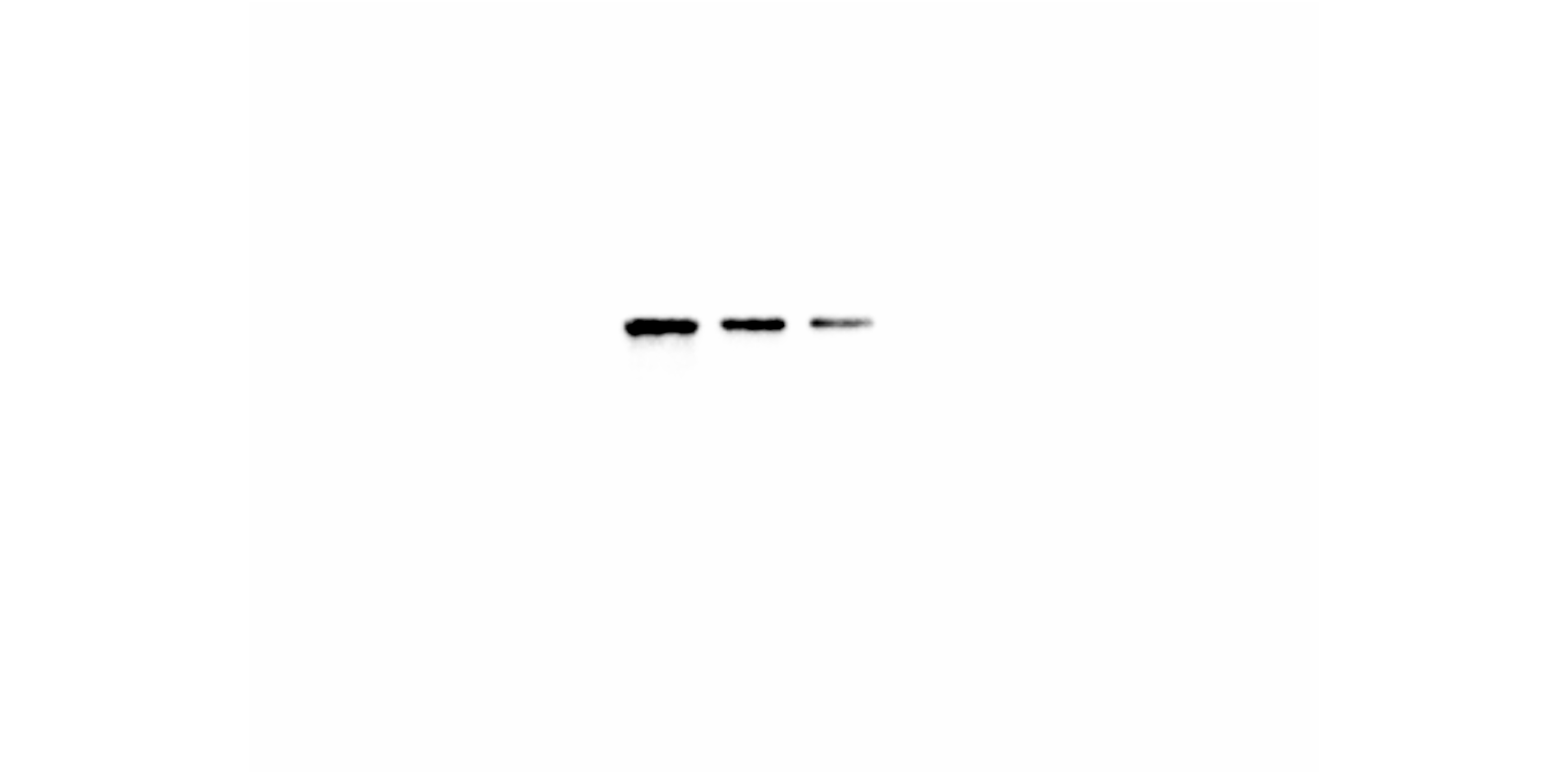

Supplement: Supplementary file 28 — Appendix S28. [file JCMM-28-e70188-s013.tif]
